# Preserved blood-brain barrier and neurovascular coupling in female 5xFAD model of Alzheimer’s disease

**DOI:** 10.3389/fnagi.2023.1089005

**Published:** 2023-05-05

**Authors:** Oleg Zhukov, Chen He, Rana Soylu-Kucharz, Changsi Cai, Andreas D. Lauritzen, Blanca Irene Aldana, Maria Björkqvist, Martin Lauritzen, Krzysztof Kucharz

**Affiliations:** ^1^Department of Neuroscience, Faculty of Health and Medical Sciences, University of Copenhagen, Copenhagen, Denmark; ^2^Biomarkers in Brain Disease, Department of Experimental Medical Sciences, Lund University, Lund, Sweden; ^3^Department of Computer Science, University of Copenhagen, Copenhagen, Denmark; ^4^Department of Drug Design and Pharmacology, Faculty of Health and Medical Sciences, University of Copenhagen, Copenhagen, Denmark; ^5^Department of Clinical Neurophysiology, Rigshospitalet, Copenhagen, Denmark

**Keywords:** Alzheimer’s disease, 5xFAD, two-photon microscopy, blood-brain barrier, adsorptive-mediated transcytosis, neurovascular coupling, *in vivo*, paracellular permeability

## Abstract

**Introduction:**

Dysfunction of the cerebral vasculature is considered one of the key components of Alzheimer’s disease (AD), but the mechanisms affecting individual brain vessels are poorly understood.

**Methods:**

Here, using *in vivo* two-photon microscopy in superficial cortical layers and *ex vivo* imaging across brain regions, we characterized blood–brain barrier (BBB) function and neurovascular coupling (NVC) at the level of individual brain vessels in adult female 5xFAD mice, an aggressive amyloid-*β* (A*β*) model of AD.

**Results:**

We report a lack of abnormal increase in adsorptive-mediated transcytosis of albumin and preserved paracellular barrier for fibrinogen and small molecules despite an extensive load of A*β*. Likewise, the NVC responses to somatosensory stimulation were preserved at all regulatory segments of the microvasculature: penetrating arterioles, precapillary sphincters, and capillaries. Lastly, the A*β* plaques did not affect the density of capillary pericytes.

**Conclusion:**

Our findings provide direct evidence of preserved microvascular function in the 5xFAD mice and highlight the critical dependence of the experimental outcomes on the choice of preclinical models of AD. We propose that the presence of parenchymal A*β* does not warrant BBB and NVC dysfunction and that the generalized view that microvascular impairment is inherent to A*β* aggregation may need to be revised.

## Introduction

Alzheimer’s disease (AD) is the most common cause of dementia, with the presence of amyloid-*β* (A*β*) plaques as the dominant marker for the neuropathological diagnosis. Deposition of A*β* is thought to cause neurovascular dysfunction, compromised brain energy supply, and ultimately, loss of cognitive performance (Iadecola, 2017; Sweeney et al., 2018a; Nation et al., 2019). The traditional view holds that the dysregulation of the blood–brain barrier (BBB) and cerebral blood flow (CBF) in AD is strongly associated with A*β* (Hartz et al., 2012; Kook et al., 2012; Iadecola, 2017; Park et al., 2017; Sweeney et al., 2018a; Nortley et al., 2019; Wang et al., 2021; Nehra et al., 2022). However, this view is met by contrasting findings showing no pathological BBB leakage both in preclinical models of AD (Klohs et al., 2013; Bien-Ly et al., 2015; Marottoli et al., 2017; Gustafsson et al., 2018; Ries et al., 2021), and in patients (Profaci et al., 2020). Consequently, the contribution of A*β* to vascular dysfunction is debatable, and a more detailed characterization of the effects of A*β* burden on cerebral vasculature is needed to clarify the issue.

Here, we used two-photon microscopy (2 PM) *in vivo* to characterize both BBB and neurovascular coupling (NVC) at distinct categories of cerebral microvessels in adult female 5xFAD mice, a commonly used AD model, characterized by an extensive A*β* plaque deposition with metabolic and behavioral deficits (Oakley et al., 2006; Andersen et al., 2021; Forner et al., 2021; Tsui et al., 2022). We found that despite a massive A*β* deposition, microvascular functions were preserved in 5xFAD mice: (i) adsorptive-mediated transcytosis (AMT) remained unchanged in all segments of pial and parenchymal vessels, (ii) paracellular permeability was unchanged, (iii) NVC was unaffected along the microvascular tree, i.e., penetrating arterioles, precapillary sphincters, and downstream capillaries, and (iv) the density of capillary pericytes were at the levels of WT mice. Together, our findings suggest that even in presence of A*β,* BBB and NVC can be well-functional in the 5xFAD model of AD.

## Materials and methods

### Animals

All procedures involving animals were approved by the Danish National Committee on Health Research Ethics in accordance with the European Council’s Convention for the Protection of Vertebrate Animals Used for Experimental and Other Scientific Purposes and followed the Animal Research: Reporting *In Vivo* Experiments (ARRIVE) guidelines. The animal housing facility has been accredited by the Association for Assessment and Accreditation of Laboratory Animal Care (AAALAC) and the Federation of Laboratory Animal Science Associations (FELASA).

We used 5xFAD mice (Tg(APPSwFlLon,PSEN1*M146L*L286V)6799Vas/Mmjax; JAX strain: 006554) and age-matched wild-type littermate (WT) mice on the mixed B6/SJLF1J background (The Jackson Laboratory, Bar Harbor, ME, USA). The colony was maintained by backcrossing to B6/SJL hybrids at the department of Drug Design and Pharmacology, University of Copenhagen. The 5xFAD mice overexpress five familial AD mutations in human APP and PSEN1 under the control of the Thy1 promoter, resulting in rapid deposition of A*β* with no deposition of tau (Oakley et al., 2006). At the age of 6 months and older, 5xFAD mice develop substantial pathology expressed as A*β* plaques, alterations in behavior, and memory deficits (Oakley et al., 2006; Jawhar et al., 2012; Forner et al., 2021).

Female 5xFAD mice exhibit more severe A*β* pathology than males (Oakley et al., 2006), thus, only females were used in the study. In total, we used 21 5xFAD and 21 WT mice. Of those, eight 8–10-month-old (m.o.) mice of each genotype were used to measure AMT; ten 7–11-m.o. 5xFAD and WT mice were used to assess NVC. Paracellular leakage was measured in six 5xFAD and seven WT mice after the measurement of NVC; one 5xFAD mouse was excluded from the analysis of paracellular leakage because of a substantial drift along the optical axis. Three 5xFAD and three WT mice (all 7-m.o.) were used for immunohistochemistry (IHC) analysis of the fibrinogen leakage and pericyte density. Genotyping of 5xFAD mice was performed from tail clippings by a standard PCR protocol (JAX protocol: 23370) for the APP gene using the following primers: transgene forward: AGG ACT GAC CAC TCG ACC AG (olMR3610), transgene reverse: CGG GGG TCT AGT TCT GCA T (olMR3611), internal positive control forward: CTA GGC CAC AGA ATT GAA AGA TCT (olMR7338), internal positive control reverse: GTA GGT GGA AAT TCT AGC ATC ATC C (olMR7339).

In addition, we used nine 6-7-m.o. male C57BL/6J mice and one 6-m.o. transgenic reporter mouse expressing cytosolic green fluorescent protein (GFP) under the endothelial-specific receptor tyrosine kinase (Tie2) promoter [heterozygous Tg(TIE2GFP)287Sato/J (Tie2-GFP) mice; The Jackson Laboratory (JAX) #003658].

All mice were housed in ventilated cages under a 12 h light/12 h dark cycle, at 50 ± 10% relative humidity, at room temperature, with *ad libitum* access to food and water.

### Surgical procedures

Mice underwent surgery as previously described (Kucharz and Lauritzen, 2018). Briefly, anesthesia was induced by intraperitoneal (i.p.) injections of xylazine (10 mg/kg) and ketamine (60 mg/kg). During surgery, mice were kept anesthetized by supplementary i.p. injections of ketamine (30 mg/kg) every 25–30 min. A 3-mm diameter craniotomy was drilled over the somatosensory barrel cortex, the dura was removed, and the opening was sealed with a lukewarm agarose solution and then covered with a coverslip. Artificial cerebrospinal fluid (aCSF) (in mM: NaCl 120; KCl 2.8; NaHCO3 22; CaCl2 1.45; Na2HPO4 1; MgCl2 0.876, and glucose 2.55; pH = 7.4) was applied to the cranial window to keep the brain moist. To allow the insertion of a recording microelectrode, an opening was left at the lateral side of the craniotomy between the rim of the skull bone and the coverslip. After surgery, the anesthesia was switched to *α*-chloralose (0.5 g/mL) infused through the venous catheter at the rate of 0.01 mL/h/10 g.

### Whisker pad stimulation

To study NVC in the somatosensory cortex, we stimulated the contralateral trigeminal nerve using custom-made bipolar electrodes, as previously described (Kucharz and Lauritzen, 2018). Briefly, the cathode was positioned relative to the hiatus infraorbitalis (IO), and the anode was inserted into the masticatory muscles. Thalamocortical IO stimulation consisted of a 15-s train of 1-ms pulses (pulse amplitude 1.5 mA) at the rate of 2 Hz (ISO-flex; A.M.P.I.). Stimulation was triggered using Spike2 software (v. 7.2.; Cambridge Electronic Design) and was synchronized with image acquisition by the two-photon microscope (Olympus FVMPE-RS, v01.02.01.27).

### Recording and quantification of cortical local field potentials

To monitor brain activation during whisker-pad stimulation, we recorded local field potential (LFP) as previously described (Jessen et al., 2015). Briefly, single-barreled borosilicate microelectrode (resistance 1.5–2.5MOhm) was inserted into the whisker-barrel cortex, and the reference electrode was placed under the skin of the neck of the mouse. The signal was first preamplified (gain 10x; low-pass cutoff frequency, 3,000 Hz; DP-311 differential amplifier, Warner Instruments), then additionally amplified and filtered (gain 100x, high-pass cutoff frequency 1 Hz; CyberAmp 320, Axon Instruments). Digital sampling was performed using a 1,401 mkii interface (Cambridge Electronic Design) connected to Spike2 software (v. 7.2.; Cambridge Electronic Design) at the sampling rate 20 kHz. We calculated the amplitude of the negative peak of the LFP (Jessen et al., 2015) and averaged the LFP amplitudes over all but the first LFP within a stimulation train.

### Two-photon microscopy and image analysis

We used a FluoView FVMPE-RS two-photon microscope (Olympus, v01.02.01.27) equipped with a tunable femtosecond laser (Mai-Tai DeepSee). The fluorescence was recorded using a 25x (NA = 1.05) water immersion objective and high-sensitivity GaAsP detectors.

#### Amyloid-*β* plaques

A*β* plaques were visible by autofluorescence (Kwan et al., 2009; Gao et al., 2019). We assessed the A*β* plaque load by calculating the density of plaques from autofluorescence images of the brain (Supplementary Video S1). To confirm that the autofluorescence originates from A*β*, we labeled the plaques in one 9-m.o. 5xFAD mouse by topical application of thioflavin-S (Sigma-Aldrich, #T1892, 0.01% in aCSF) for 20 min. Thioflavin-S was replaced with aCSF before image acquisition. Both autofluorescence and thioflavin-S were excited at 800 nm, and the emitted light was registered in the range of wavelengths below 560 nm. The overlap between thioflavin-S-positive plaques and the autofluorescence confirmed the identity of the plaques observed without labeling.

The density of A*β* plaques in the cortex was calculated from Z-stack images (250 μm × 250 μm × 150 μm) of cerebral cortex per mouse as the number of plaques divided by the volume of the brain in one Z-stack. The plaques were counted manually using ImageJ [v2.3.0 NIH (Schindelin et al., 2012)]. The brain volume was determined as the volume enclosed between the brain surface and the bottom of the Z-stack. The location of the brain surface was determined by manual labeling of the surface using the ImageJ point tool (50–200 points), followed by linear interpolation between the points.

#### Adsorptive-mediated transcytosis

We quantified AMT as previously described (Mathiesen Janiurek et al., 2019), with modifications. Following injection of bovine serum albumin-Alexa Fluor 488 conjugate (BSA-Alexa488) (5xFAD mice) or BSA-Alexa594 (Tie2-GFP mice) [Thermofisher #A13100 and #A13101; 5 mg/mL, 100 μL/30 g; bolus intraarterial (i.a.)], a time-lapse Z-stack (hyperstack; 250 μm × 250 μm × 150 μm, pixel size 0.124 μm, Z-step 2.5 μm) was recorded from the barrel cortex were recorded for over 120 min. In the experiments with Tie2-GFP mice, the excitation wavelength was 800 nm, and the emitted light was recorded in two channels: 489–531 nm (GFP) and 601–657 nm (BSA-Alexa594). In experiments with 5xFAD mice, the excitation wavelength was 920 nm, and the same cutoff wavelength was used to record the emitted light of BSA-Alexa488 and autofluorescence.

AMT was quantified in the following vessel types, which were identified based on their morphology, branching pattern, and location (Mathiesen Janiurek et al., 2019; Grubb et al., 2020; Kucharz et al., 2021): pial arterioles, penetrating arterioles in pia, penetrating arterioles in the brain parenchyma, capillaries, ascending venules in the brain parenchyma, ascending venule in pia and, pial venules. Because BSA-Aexa forms punctae at the BBB interface, indicating AMT (Mathiesen Janiurek et al., 2019), we quantified AMT by calculating the density of BSA-Alexa punctae at specific vessel types. For segment i of vessel type typ, we counted the number of punctae, $n_{i,typ}$ and calculated the segment surface area from the length and the diameter of the segment, $a_{i,typ}=diameter_{i,typ}*length_{i,typ}*\pi$. ImageJ was used to manually count the punctae and measure the length and the diameter of vessel segments. For each mouse, we calculated punctae density at a specific vessel type $\rho_{typ}=\sum_{i}n_{i,typ}/\sum_{i}a_{i,typ}$. Overall punctae density was calculated by dividing the total number of punctae by the total surface area in all vessel segments: $\rho_{overall}=\sum_{i,typ}n_{i,typ}/\sum_{i,typ}a_{i,typ}$. Because of a limit in the width of recorded Z-stacks, some vessel types were missing in a Z-stack; therefore, the number of data points of those vessel types was smaller than the number of mice.

#### Paracellular leakage

We quantified paracellular leakage by measuring the intensity of sodium fluorescein (NaFluo, 376 Da) in the brain parenchyma as previously described (Mathiesen Janiurek et al., 2019), with modifications. Following the injection of NaFluo (10 mg/mL, 0.05 mL/30 g; bolus i.a.), we recorded time-lapse Z-stack images (250 μm × 250 μm × 150 μm, pixel size 0.124 μm, Z-step 4 μm, time step 3 min, total recording time 45 min) spanning from the brain surface down to ∼100 μm below the brain surface. The time frame of 45 min was chosen based on our previous study demonstrating increased NaFluo leakage in inflammatory-like conditions (Mathiesen Janiurek et al., 2019). The excitation wavelength was 800 nm, and the emitted light was recorded within the 489–531 nm band.

The intensity of NaFluo in parenchyma was quantified at 20, 40, and 80 μm below the brain surface by averaging the signal within 4–5 regions of interest (ROI) at each depth. The depth was calculated relative to the brain surface for each ROI individually, so the difference in depths between the ROIs is less than the Z-step. The NaFluo fluorescence in parenchyma was heterogenous, likely due to absorption of the fluorescence light by big pial vessels and by A*β* plaques (Supplementary Figures S1A–B; Supplementary material, section 1). We minimized the effect of the heterogeneity on our quantification by avoiding placing ROIs under blood vessels and under A*β* plaques (Supplementary material, section 1). The NaFluo intensity in the blood was averaged within ROIs placed in a pial vessel. Lateral drift was corrected for all ROIs by calculating the translation from the maximum-intensity projected images and shifting the position of the ROIs according to the translation. We quantified paracellular leakage as the ratio of the area under the curve (AUC) of the intensity in the parenchyma and the AUC of the intensity in the blood. This quantity takes into account differences in the intensity in the blood between WT and 5xFAD (Supplementary Figure S1C). One dataset was excluded from the analysis because of a substantial drift along the optical axis.

#### Neurovascular coupling

NVC was assessed by quantifying vasodilation of penetrating arterioles, precapillary sphincters, 1st-, and 2nd-order capillaries upon whisker pad stimulation. We classified the vasculature by the branching order: zero-order being the penetrating arteriole, first-order being the primary capillary branching off the arteriole, with a consecutive increase in branching order corresponding to downstream branching of capillaries (Hall et al., 2014). We characterized the precapillary sphincter as an indentation of the capillary where it emerges from the penetrating arteriole (Grubb et al., 2020). We labeled vessel lumen with intravascular dye (TexasRed 70-kDa dextran, 20 mg/mL, 0.05 mL/30 g; bolus i.a.) and recorded a time-lapse of Z-stacks capturing the vessels before, during, and after stimulation. Time-lapse Z-stack ensures that focal drift does not affect the diameter quantification by scanning the vessels at a range of depths at each time point. A single Z-stack consisted of 9–14 focal planes separated by 2.5 μm; 1–2 s per Z-stack. A single time-lapse recording lasted ∼two minutes and consisted of a 40-s baseline, 15-s stimulation, and a 75-s post-stimulation period. Imaging was performed in 2–5 locations per mouse at depths of 50–250 μm. The locations were selected based on the following criteria: (i) the penetrating arteriole is responsive upon stimulation; (ii) the downstream capillaries can be clearly visualized, i.e., they are not positioned under the penetrating arteriole, which reduces the contrast of structures underneath. The selection of vessels was not influenced by the presence or the location of A*β* on the arterioles. Each location was imaged 2–3 times at five-minute intervals. The excitation wavelength was 920 nm, and the emitted light was registered within the 601–657 nm band. In order to achieve better temporal resolution, fast resonant scanning was used.

Vasodilation was quantified using custom-made MATLAB software as previously described (Cai et al., 2018; Zambach et al., 2021). In brief, to quantify vessel diameter, the 2 PM hyperstack images were maximum-intensity projected along the optical axis. If needed, specific focal planes were selected in which the vessel was clearly visualized. A rectangular ROI was drawn across the vessel’s longitude. The average profile of the vessel was obtained from the ROI at each time frame. A threshold was manually adjusted to produce a mask of the vessel profile over time (Cai et al., 2018). The threshold was kept the same in time and among ROIs drawn on the same vessel type and recording. The vessel diameter was calculated as the number of foreground pixels in the mask at each time frame, multiplied by the pixel size. The baseline diameter, *D*_0_, was calculated as average over all frames before the onset of stimulation. A diameter trajectory was excluded if (i) the baseline diameter was largely unstable or (ii) the spontaneous dilation began before the stimulus onset. The peak diameter, *D_p_*, was estimated as the maximum of the smoothed (running average, window size of five points) diameter trajectory between the stimulus onset and 40s thereafter (Cai et al., 2018). The relative dilation was calculated as (*D_p_*–*D*_0_)*/D*_0_.

### Administration of lipopolysaccharide

To test our AMT protocol under controlled conditions, we measured AMT in mice with acute systemic inflammation, which is accompanied by an increase in AMT (Banks and Erickson, 2010; Wang et al., 2019). Inflammation was induced in three C57BL/6 J mice by administering 5 mg/kg i.p. lipopolysaccharide (LPS) (500X lipopolysaccharide from *E. coli* O111:B4; #00–4976-93, Thermofisher) (Qin et al., 2007). Because LPS triggers the release of proinflammatory factors into serum within 1–2 h (Tateda et al., 1996; Qin et al., 2007), we injected BSA-Alexa488 one hour after LPS administration and quantified AMT two hours after injection of BSA-Alexa488.

### Quantification of adsorptive-mediated transcytosis by machine learning

We generated two U-net-based models (Ronneberger et al., 2015) to quantify AMT automatically. Model-1 takes as input a single image, consisting of fluorescence and autofluorescence channels, and generates a binary mask of the blood vessel; Model-2 receives the same input as Model-1 and generates a binary mask of BSA-Alexa488 punctae (Supplementary Figure S2). The surface area of the vessel was calculated from the vessel mask as the number of vessel pixels times pixel area (0.124 × 0.124 μm^2^) multiplied by *π*, and the number of albumin punctae was calculated from the punctae mask. As in the manual analysis, the density of the punctae at each type of vessel was calculated as the total number of punctae divided by the total surface area obtained from all ROIs of this vessel type.

For the model training and validation, 77 ROIs of pial arterioles and pial venules were cut from the 2 PM hyperstack data recorded in two 5xFAD and three WT mice. Each ROI was maximum intensity projected along the optical axis resulting in a set of five frames per ROI and two channels: one channel captured fluorescence from BSA-Alexa488, and another channel captured autofluorescence of the brain tissue. By using both channels, Model-2 trained to recognize BSA-Alexa488 punctae but not autofluorescence (Supplementary Figure S3). The total number of projections for each channel was 385. BSA-Alexa488 punctae and blood vessels were manually annotated by drawing binary segmentation masks, which were used as ground truth during the training. Sixty-five ROIs (325 projections) were randomly allocated for the training of the models, and the remaining 12 ROIs (60 projections) were allocated for validation. The training and validation data were independent, in terms of not overlapping, with the data used to quantify AMT.

Model-1 and Model-2 were trained independently from each other. The training maximized the Dice similarity coefficient (DICE) until convergence in the validation set (Supplementary Figure S4). DICE was defined as follows (Dice, 1945; Asgari Taghanaki et al., 2021):


(1) $$DICE\left(\overset{\wedge }{y},y\right)=\frac{2\sum \left(\overset{\wedge }{y}\times y\right)}{\sum \overset{\wedge }{y}+\sum y},$$

where *y*ˆ and *y* are the prediction and the ground-truth segmentation masks, respectively, and *y*ˆ × *y* is the element-wise multiplication of the predicted and ground-truth masks. The sums are calculated over all the indices in the masks. The DICE coefficient was chosen over other objectives to accommodate the large class imbalance between background and punctae pixels. The punctae and vessel segmentation models were trained for 200 and 100 epochs, respectively.

During training, the input projected ROIs were augmented by random rotating, scaling, shearing, contrasting, brightening, and transposing (Shorten and Khoshgoftaar, 2019).

### Immunohistochemistry

Mice were anesthetized with Xylazine/Ketamine and transcardially perfused for 1–2 min with phosphate-buffered saline (PBS) at the rate of 10 mL/min, then for 4–5 min with ice-cold 4% paraformaldehyde (PFA) in PBS. Next, the brains were post-fixed in 4% PFA for 24 h at 4°C and subsequently cryoprotected in 25% sucrose and 0.1% sodium azide in PBS for 24 h at 4°C. The brains were frozen in dry ice and sectioned into 30 μm thick coronal sections using a sliding microtome Microm HM450 microtome (ThermoFisher Scientific).

To perform fibrinogen staining, we carried out antigen retrieval in Tris/EDTA buffer (pH9.0) for 30 min at 80°C, followed by a 20-min quenching step in 3% H_2_O_2_ and 10% methanol in Tris-buffered saline (TBS). The sections were then washed 3x in TBS and pre-incubated with 5% normal goat serum in 0.25% triton-X in TBS (TBS-T) for 1 h at room temperature (RT). Next, the sections were incubated overnight at RT with primary antibody anti-fibrinogen (1:2000, ab227063, Abcam) in 5% normal goat serum TBS-T solution. After the washing step of 3x in TBS-T for 10 min each, the sections were incubated in 1% BSA in TBS-T with a biotinylated secondary goat-anti-rabbit antibody (1:200; Vector Laboratories Inc.). Next, the sections were rewashed 3x in TBS-T for 10 min each, followed by incubation with an avidin-biotin-peroxidase complex solution for 1 h at RT, and washed 3x again in TBS-T for 10 min each. Finally, the staining was visualized using 3,3′-diaminobenzidine (DAB) and 0.01% H_2_O_2_ according to the manufacturer’s instructions. The sections were then mounted on chromatin-gelatin coated glass slides, stained in 0.1% cresyl violet solution for 3 min, dehydrated in increasing alcohol solutions (70, 95, 100%), cleared in xylene, and coverslipped using DPX mountant (Sigma-Aldrich) for imaging.

For the confocal imaging, after the initial washing step from the anti-freeze solution, the brain sections were preincubated with 5% normal donkey serum in PBS with 0.3% Triton-X-100 for 1 h at RT. The sections were next incubated overnight at RT in PBS with 0.025% Triton-X-100 (PBST) with 3% normal donkey serum solution containing primary antibody anti-CD31 (1:400, BD Biosciences, BD550274). After washing the sections with PBS 3x, an antigen retrieval step was carried out in citrate buffer (pH6) for 30 min at 80°C. Next, the sections were washed again 3x for 10 min in PBS, then incubated overnight at RT with the primary antibody anti-PDGFR-*β* (1:100, Cell Signaling #3169). Subsequently, the sections were rinsed in PBST and incubated with Cy3 (1:500; donkey anti-rat, Jackson ImmunoResearch, #712–165–153) and AlexaFluor 647 (1:500, donkey Anti-Rabbit, ab150075) secondary antibodies, diluted in 10% normal serum in PBST for 1 h at RT. Next, the sections were rinsed 3x for 10 min in PBST and stained with DAPI for 10 min, followed by a washing step in PBS. Finally, the brain sections were mounted on gelatin-covered glass slides and coverslipped using an antifading mountant (ProLong Diamond Antifade Mountant, Thermofisher Scientific, #P36965).

### Bright-field imaging

We used bright-field imaging to assess fibrinogen extravasation. The imaging was performed using BX53 upright transmitted light microscope (Olympus), equipped with 4 × 0.16NA UPLSAPO objective for collecting section overview images and 20 × 0.75NA UPLSAPO super apochromat objective for blood vessel imaging. The light was collected by a DP73 camera with an exposure time of 5.9 ms at ISO 200 and in 24-bit sRGB Color mode with the same microscope settings between all sections and genotypes.

### Fibrinogen quantification

We assessed the fibrinogen leakage by quantifying both the average fibrinogen within a set of defined brain regions and at the proximity of the brain vessels. First, the sRGB images were split in ImageJ into the channels with the highest specificity toward absorbance of cresyl violet (red channel), intermediate signal (green channel), and fibrinogen (blue channel). Next, the fibrinogen channel was inverted, so the increase in pixel intensity corresponds to the increase in the fibrinogen in the tissue [channels marked fib. (inv.)].

The overall level of fibrinogen in the brain was quantified from the low magnification overview images by averaging the signal within ROIs covering different brain regions: cerebral cortex, hippocampus, and hippocampal subregions (stratum lacunosum-moleculare (s.l-m) and the remaining hippocampal areas).

The local vessel-associated fibrinogen was quantified from the high-magnification images by comparing the intensity profile across the vessel wall between WT and 5xFAD. The vessel boundary was manually traced using the segmented line tool (ImageJ). A region around the trace (3 μm toward lumen and 11 μm toward parenchyma) was extracted and straightened into a rectangle image with dimensions of L (=vessel length) and x (= constant width). To minimize the interference of the cresyl violet nuclei labeling on the measured signal of fibrinogen, we manually set a threshold mask that encompassed nuclei and removed these pixels from the corresponding channel with fibrinogen. Subsequently, the data was projected along the L-axis to calculate the average signal profile across the vessel. This procedure has been performed for both sides of each vessel. For each animal, a weighted average profile was calculated to account for the different lengths of sampled vessels.

### Confocal imaging

We performed confocal fluorescence imaging to quantify pericytes on the cortical microvasculature. The images were collected using DMi8 inverted fluorescence microscope (Leica Microsystems) equipped with HC PL APO 40 × 1.3NA oil-immersion objective. We used 405 nm, 552 nm, and 638 nm laser diodes to excite DAPI (nuclei), Cy-3 (endothelium), and AlexaFluor 647 (pericytes), and the emitted fluorescence was collected by a hybrid detector after 440–480 nm; 565–585 nm; and 655–685 nm bandpass, respectively. The data was collected as Z-stacks with triple line averaging and at the resolution of 1,024 × 1,024 pixels and Z-step = 2 μm, corresponding to the volume of 388 μm × 388 μm with average stack depth = ∼26 μm. To minimize the bleed-through between the channels, the imaging was performed for each channel separately, i.e., in sequential mode. In addition, we used a 488 nm excitation wavelength to collect the signal after a 655–685 nm bandpass, obtaining an autofluorescence image of the brain tissue. The images were exported to ImageJ as multi-channel .tiff files for further analysis.

### Pericyte quantification

We quantified the number of pericytes per unit length of capillaries. Ten Z-stacks were acquired from the somatosensory cortex (2–3 mm posterior from bregma) in 3–4 sections per animal. First, we subtracted the autofluorescence signal from the CD31 imaging channel. The vessel lengths were calculated manually using ImageJ. Only vessels that corresponded to the diameter of capillaries (*<*10 μm) were considered. Next, we counted pericyte cell bodies with the inclusion criteria: (i) the presence at the microvessel and (ii) typical “bump on a log” pericyte morphology with a clear DAPI signal in nuclei. For each Z-stack, we calculated the pericyte density as the number of capillary-associated pericyte cell bodies divided by the total length of the capillaries.

### Statistical analysis

Statistical analysis was performed using *Scipy* 1.9.1 (Virtanen et al., 2020) in Python 3 (Van Rossum and Drake, 2009). Linear regression was performed using Python *statsmodels* 0.13.5 (Seabold and Perktold, 2010). Data are presented as mean ± standard error of the mean (SEM) unless otherwise stated. Statistical tests were performed on averages within individual mice, i.e., on statistically independent data. The sample sizes were based on estimates from our previous research (Mathiesen Janiurek et al., 2019; Zambach et al., 2021). Normality was tested by using the Shapiro–Wilk test. Differences between groups were tested by Student’s *T*-test or Mann–Whitney U test for normally and non-normally distributed data, respectively. The outcome of the tests was assessed at a significance level of 0.05. We avoided outlier testing and elimination to prevent inflation of the type-I error rate (see Supplementary material, section 4 for details). Descriptive and test statistics, as well as *p*-values, are listed in Supplementary Table S2. Blinded quantification of paracellular leakage and AMT was impossible because it involved manual placement of ROI or counting of BSA punctae, during which the autofluorescence of A*β* plaques was visible. Fibrinogen image collection and data analyses were performed by an experimenter blinded to the experimental conditions. Analysis of LFP and NVC was automated and blinded toward the genotype. The blinding was not possible during pericyte IHC assessment due to the clear presence of the A*β* aggregates on the fluorescence images in 5xFAD mice.

## Results

### *In vivo* two-photon imaging in 5xFAD mice

We characterized the BBB and NVC in 7–11-m.o. 5xFAD mice using 2 PM *in vivo* at cortical depth up to 250 um. 5xFAD is a widely used AD model because of a widespread A*β* plaque deposition, starting at 2 months (Oakley et al., 2006; Forner et al., 2021).

**Figure 1:**
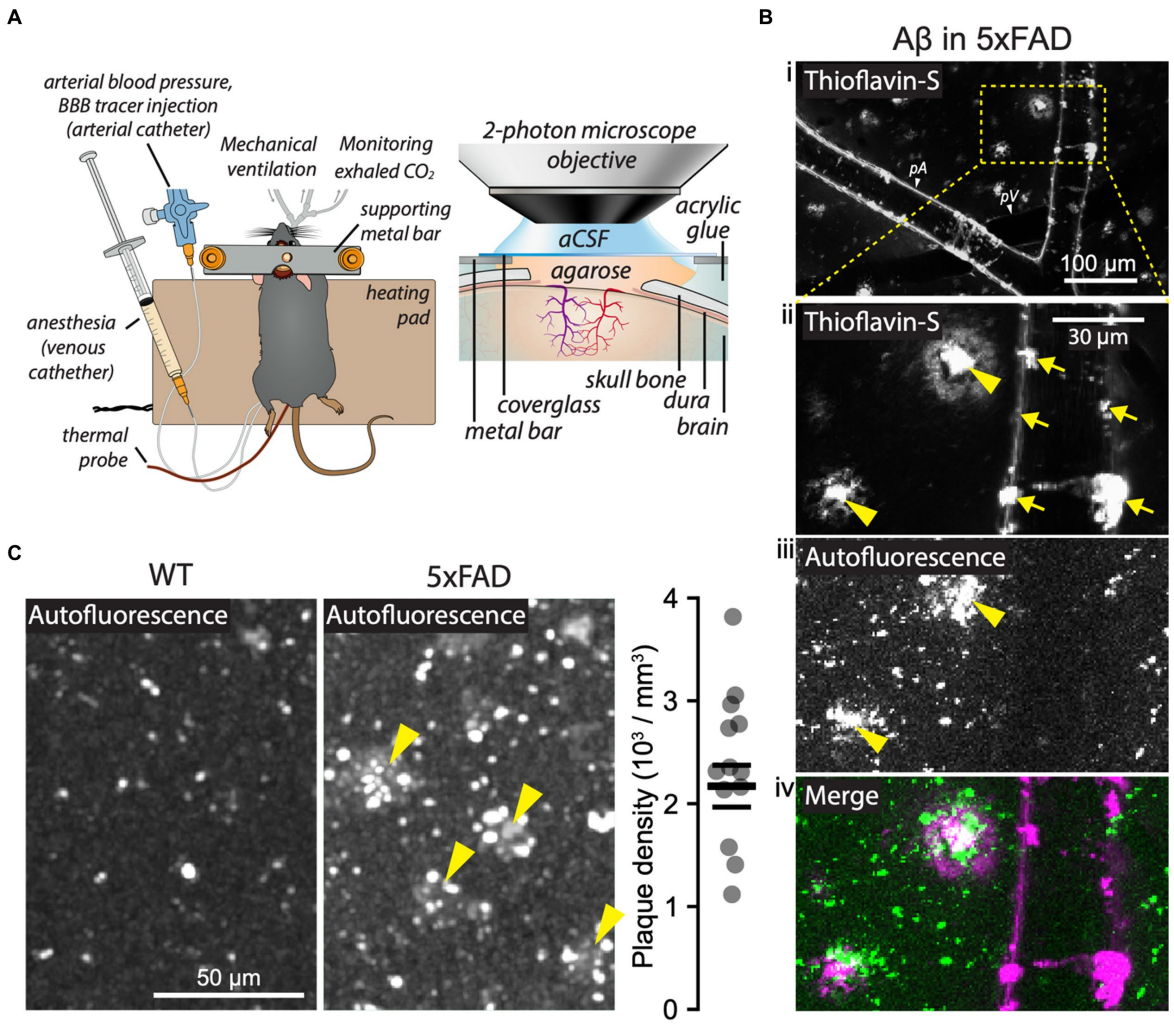
Two-photon microscopy (2 PM) in 5xFAD mice *in vivo*. **(A)** Schematic of an anesthetized mouse after tracheotomy, catheterization of femoral vessels, and craniotomy (*left*), with detailed features of the cranial window for 2 PM imaging (*right*). **(B)** A*β* visualized in a 5xFAD mouse by thioflavin-S labeling (Bi, Bii, Biv; 0.01% w/v in aCSF; 20 min of topical application) and by autofluorescence (Biii, Biv). Thioflavin-S labeled both parenchymal A*β* plaques (Bii, yellow arrowheads) and vessel-associated A*β* (Bii, arrows). A*β* plaques were also detected by autofluorescence (Biii, arrowheads). Note that the overlap between the autofluorescence and the thioflavin-S-labeled structures confirms the identity of parenchymal A*β* plaques (Biv). **(C)** Representative images of brain parenchyma showing A*β* plaques (arrowheads) in 5xFAD mice and quantification of plaque density based on autofluorescence signal (5xFAD mice, *n* = 13). In panel (Bi): pA = pial arteriole, pV = pial venule. Vessels were identified by their unique morphological and 2 PM signal features (see Methods). Images in panel **(B)** are maximum intensity projections of a Z-stack spanning from brain surface down to 35 μm into the brain parenchyma. See also the full-depth Z-stack in Supplementary Video S2. Panel **(A)** was adapted from Kucharz et al. (2021) under CC-BY 4.0 license.

The mice underwent microsurgical preparations to monitor and maintain optimal systemic parameters during 2 PM imaging (Figure 1A). First, we ascertained the presence of A*β* plaques in the brain parenchyma by their autofluorescence (Kwan et al., 2009; Gao et al., 2019), and further confirmed their identity by *in vivo* labeling with thioflavin-S (Figure 1B; Supplementary Videos S1, S2). The A*β* plaques were visible in 5xFAD mice at all cortical depths, and the mean density of the plaques was 2,217 ± 238 plaques/mm^3^ (Figure 1C), consistent with previous reports (Giannoni et al., 2016; Yin et al., 2022; Supplementary material, section 2).

### Adsorptive-mediated transcytosis is unchanged in 5xFAD mice

Next, we asked whether A*β* pathology in 5xFAD mice is associated with disinhibition of AMT, a vesicular transport of blood-borne molecules (e.g., albumin) across the BBB. The increase in AMT is evidenced in aging, neuroinflammation, and stroke and is suggested to underlie and accelerate neurodegeneration (Knowland et al., 2014; Sadeghian et al., 2018; Yang et al., 2020). Yet, AMT remained unstudied in AD.

**Figure 2:**
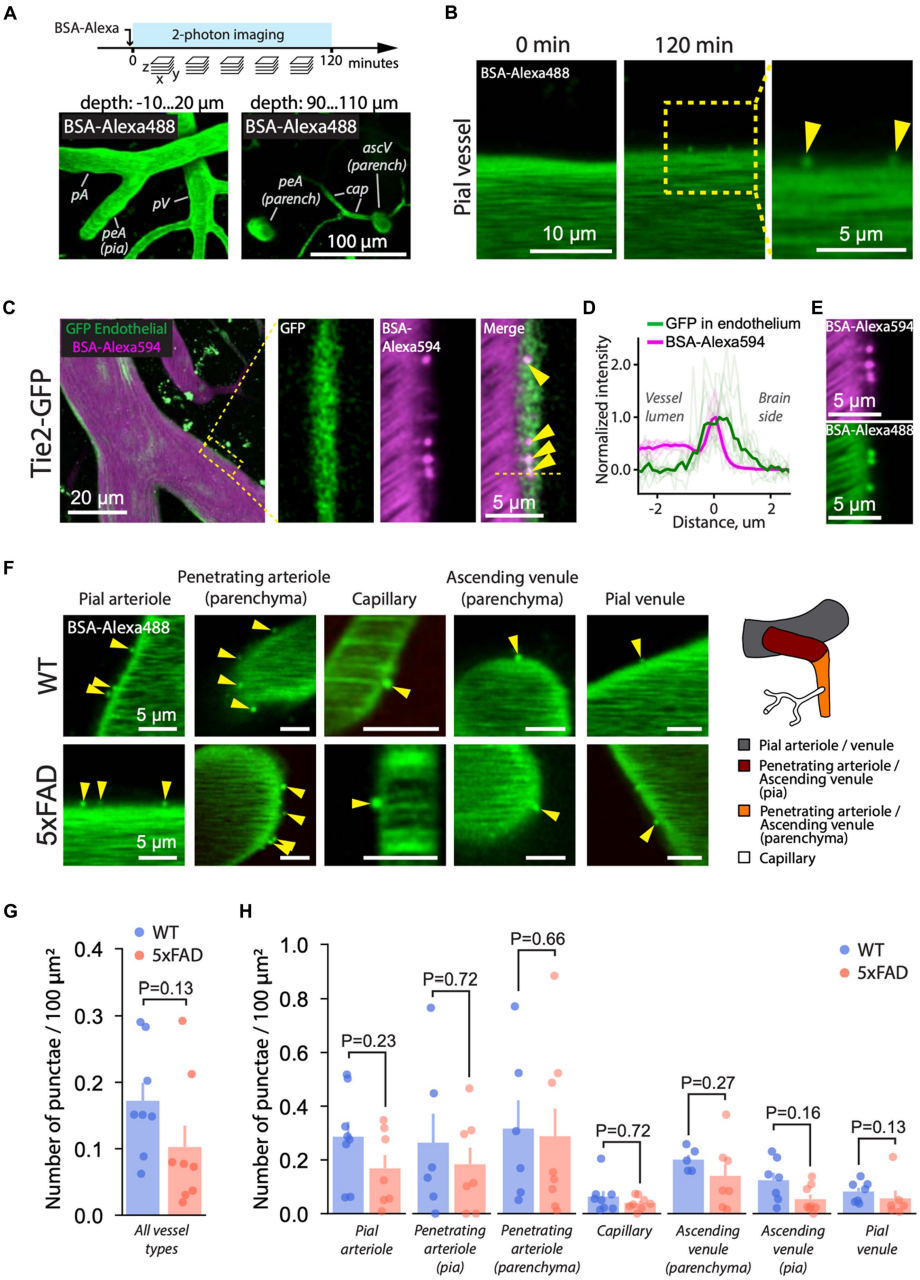
AMT is unchanged in 5xFAD mice. **(A)** Experimental timeline. Following the injection of BSA-Alexa488 (5xFAD mice) or BSA-Alexa594 (Tie2-GFP mice), fluorescence images of the brain were recorded as a series of Z-stacks over time. Z-stacks contained brain parenchyma and pial (bottom-left) and parenchymal (bottom-right) vasculature. **(B)** Following the injection, BSA-Alexa488 formed punctae (arrowheads) at the BBB interface, indicating transcytotic vesicles. The inset indicates that the images are single focal planes. **(C)** To confirm the endothelial origin of the punctae, BSA-Alexa594 was administered to a Tie2-GFP mouse expressing GFP in the endothelium. *Left*: Pial arteriole with circulating BSA-Alexa594 (red) and GFP in the endothelium (green). The green signal from outside the vessel originates from brain autofluorescence (see also Supplementary Video S1). *Right*: a zoomed-in cross-section (single focal plane) of the arteriole wall (rectangle on the left) showing the signal from the GFP, BSA-Alexa594 punctae, and the merge. The overlap between the GFP and the punctae shown in the merge and as a profile in **(D)** confirms the endothelial origin of the puncate. **(D)** Fluorescence intensity profile across punctae and the endothelium confirms the localization of punctae. Thick lines show the average profile calculated from 10 profiles (transparent lines) across individual punctae and the endothelium in a Tie2-GFP mice. **(E)** BSA formed punctae regardless of the fluorescent label, i.e., AlexaFluor 488 or AlexaFluor 594. **(F)** Two hours following the injection, BSA-Alexa488 punctae were present in all vessel types in both 5xFAD and WT mice. **(G)** Quantification of AMT across all vasculature revealed no difference between WT and 5xFAD (two-tailed Mann–Whitney U test; each dot represents an individual mouse, *n* (WT) = 8 *n* (5xFAD) = 8 mice). **(H)** Likewise, AMT was the same in WT and 5xFAD in specific vessel types (twotailed Mann–Whitney U test; each dot represents a vessel type of individual mouse). Images in **(B,E,F)** are single focal planes. Images in **(A)** are maximum intensity projections over several focal planes spanning the indicated range of depths. Images in **(C)** are a projection (left) and single focal planes (three images on the right).

To monitor AMT, we injected fluorescently labeled BSA-Alexa488 into the bloodstream (Knowland et al., 2014; Mathiesen Janiurek et al., 2019) and continuously recorded Z-stack images capturing all types of cerebral blood vessels, i.e., pial and parenchymal arterioles and venules, and capillaries for 120 min (Figure 2A). Following the injection, we observed a gradual formation of BSA punctae at the BBB, indicating vesicular uptake of circulating BSA-Alexa488 into the endothelium (Figure 2B; Supplementary Video S3). The punctae moved substantially along the BBB interface, indicating that they represent free vesicles rather than vesicular pits attached to the endothelial membrane (Supplementary Videos S4, S5). The localization of the punctae to the endothelium was additionally confirmed by overlapping fluorescence signals from GFP-labeled endothelium and BSA-Alexa594 punctae in Tie2-GFP mice (Figures 2C,D). BSA formed punctae irrespective of the type of conjugated fluorophore, i.e., AlexaFluor 488 or AlexaFluor 594 (Figure 2E).

The AMT was observed in both 5xFAD and WT mice (Figures 2F–H), and it was more prominent in arterioles than in capillaries and venules (Supplementary Figure S5), which is consistent with earlier reports (Mathiesen Janiurek et al., 2019), and with the expression of caveolin-1 that forms AMT transport vesicles (Chow et al., 2020). However, AMT was the same in WT and 5xFAD, both at the level of the whole microvascular tree (Figure 2G) and at the level of corresponding vessel types (Figure 2H). AMT is also known to increase with age (Yang et al., 2020). To test whether the differences in age within experimental groups (7–11 m.o.) influenced our AMT results, we assessed age-related changes in AMT within both WT and 5xFAD experimental groups. The age span of 4 months within the animal groups had no effect on AMT in all vessel types except penetrating arterioles; however, the slope of AMT versus age in penetrating arterioles was not different between WT and 5xFAD mice (Supplementary Figure S6A).

To control our method of AMT quantification, we exposed C57BL/6J mice to LPS, which induces neuroinflammation and a rise in AMT (Marchiando et al., 2010; Wang et al., 2019). Expectedly, LPS-treated mice exhibited AMT increase, specifically in pial arterioles and venules (Supplementary Figure S7). Noteworthy, the AMT level in the control C57BL/6 J (Supplementary Figure S7) was lower than in the WT mice (Figure 2), which may be related to the younger age of the C57BL/6 J mice (Yang et al., 2020) or to the difference in the genetic backgrounds. Overall, AMT was unchanged in 5xFAD mice, and the lack of differences between WT and 5xFAD mice is unlikely caused by a sensitivity limit of our *in vivo* assay system.

**Figure 3:**
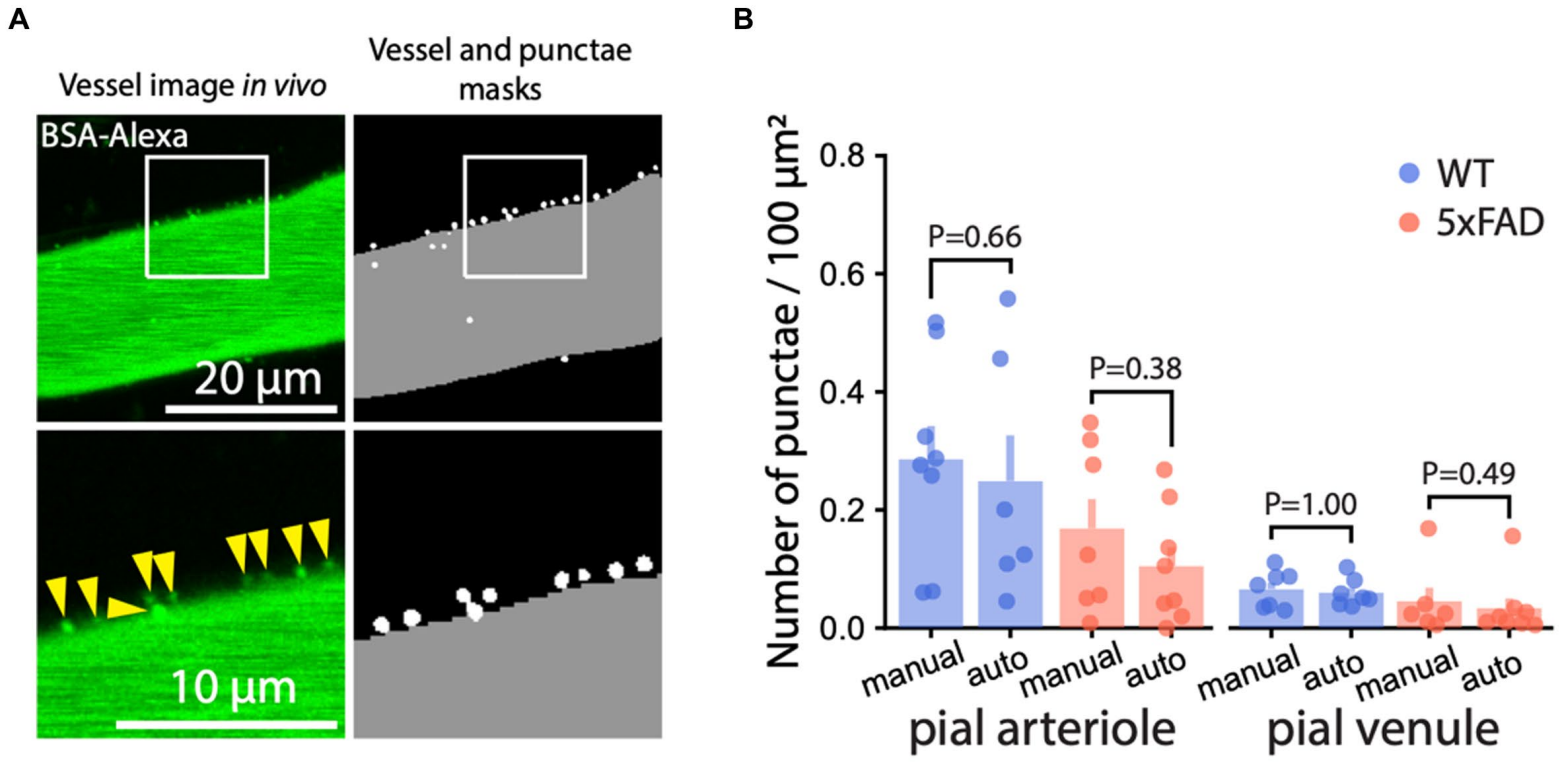
Machine learning-based quantification of AMT. **(A)** Representative example of automated segmentation of BSA-Alexa488 punctae and the vessel by machine learning (machine learning) algorithm (see Methods). *Left*: 2 PM image of a pial vessel with BSA-Alexa488 punctae; *right*: overlay of the masks locating the vessel (gray) and the punctae (white). The bottom panels show enlarged the regions within the white rectangles. **(B)** Machine learning-based quantification of AMT in pial arterioles and venules was consistent with the manual quantification (Mann–Whitney *U* test at the significance level 0.05). Images in **(A)** are maximum intensity projections.

To support our findings, we implemented a machine learning algorithm. Automatic quantification of AMT by machine learning is more objective than manual quantification and provides a measure less prone to human bias. We designed two machine learning models: Model-1 to detect vessels and Model-2 to detect BSA-Alexa488 punctae. We used the two models in conjunction to automatically quantify AMT (Figure 3; Supplementary Figures S2, S4). The performance of the models was measured by the DICE coefficient [estimates the similarity between punctae detected by the model and punctae labeled by the user (see Methods)], which reached 0.98 for Model-1 and 0.68 for Model-2 [DICE ranges between 0 (no similarity) and 1 (perfect similarity)]. Because of the small size of punctae, DICE = 0.68 was regarded as satisfactory for the counting (Asgari Taghanaki et al., 2021). Punctae density quantified by our model tended to be smaller in arterioles than the manual estimates. This trend was present in both genotypes (Supplementary Figure S8) and could be explained by too dim puncta signal for model recognition. Overall, AMT quantified by manual and automated approaches was consistent (Figure 3B), and both methods showed that AMT was unchanged in 5xFAD mice.

### Paracellular leakage across the BBB is not altered in 5xFAD mice

**Figure 4:**
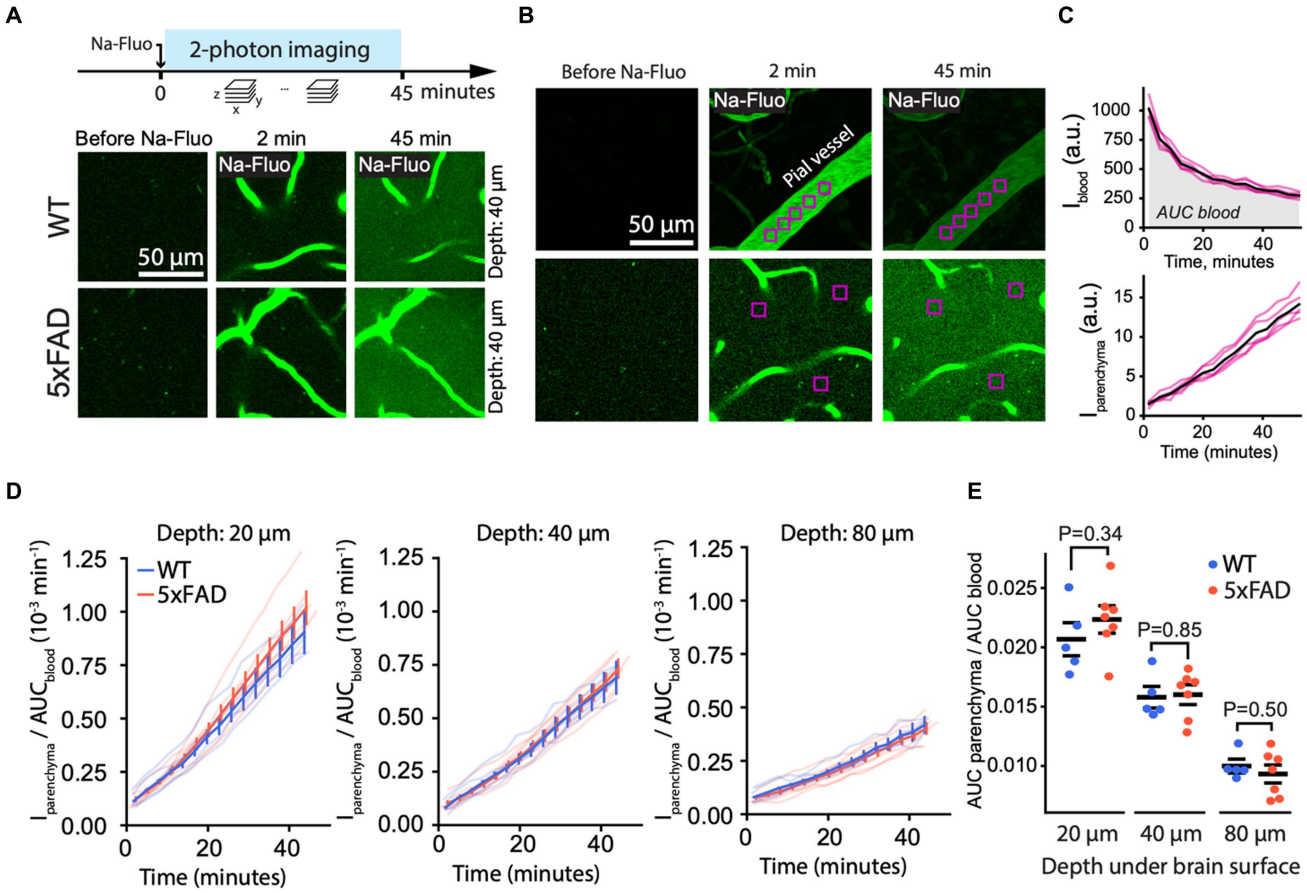
Paracellular leakage is unchanged in 5xFAD mice. **(A)** Experiment timeline (top) and representative 2 PM images of brain parenchyma (40 μm under brain surface) before and after a bolus injection of NaFluo (bottom) in WT and 5xFAD mice, showing no overt differences between the genotypes. **(B)** To quantify paracellular leakage, NaFluo intensity was calculated in the blood (top) and in the brain parenchyma (bottom) by averaging the signal within manually placed ROIs. **(C)** An example of NaFluo dynamics in blood and in parenchyma. AUC of the intensity in the blood was calculated to normalize the intensity in the parenchyma. Black curves are averages of the magenta curves representing individual ROIs. **(D)** Paracellular leakage of NaFluo into brain parenchyma expressed as the normalized NaFluo intensity at different depths under the brain surface in WT and 5xFAD mice. Thick curves are mean ± SEM calculated from curves of individual mice (transparent curves). **(E)** Quantification of paracellular leakage as AUC of the normalized NaFluo intensity in parenchyma, shown in panel **(D)**, revealed no difference between 5xFAD and WT (Student’s *T*-test; significance level 0.05; *n* (5xFAD) = 7; *n* (WT) = 5 mice). Images in **(A)** are average intensity projections over three focal planes spanning 7.5 um at indicated depth. Images in **(B)** are single focal planes.

An increase in paracellular leakage represents another mechanism of BBB dysfunction which can be studied by 2 PM (Mathiesen Janiurek et al., 2019). Accordingly, to study paracellular leakage, we injected into the mouse bloodstream sodium fluorescein (NaFluo), a small (376 Da) tracer molecule, which crosses the BBB between brain endothelial cells, i.e., *via* the paracellular route (Figure 4). Immediately after injection, we recorded fluorescence of blood-circulating and parenchymal NaFluo in time series of Z-stacks containing both pial and parenchymal vessels (Figures 4A,B). We quantified the NaFluo in the blood and its accumulation in the brain tissue by averaging the intensity within ROIs placed in pial vessels and in parenchyma at the constant, defined depth (Figures 4B,C). Following the injection, NaFluo gradually appeared in the brain parenchyma in both 5xFAD and WT due to paracellular leakage (Figure 4C; Supplementary Video S6; Supplementary material, section 3). Because the intensity of NaFluo circulating in the blood differed between WT and 5xFAD (Supplementary Figure S1), we normalized the intensity in the parenchyma by signal intensity in the blood (Figure 4D). We quantified the paracellular leakage as the ratio of the AUC of NaFluo in the parenchyma and in the blood. We found that the paracellular leakage was the same in 5xFAD and WT mice, regardless of the cortical depth (Figure 4E). In addition, paracellular leakage did not change significantly with the age of mice used in this study (Supplementary Figure S6B). Likewise, we observed no overt changes in the density of capillaries between WT and 5xFAD, which could affect the number of sources for the NaFluo to enter the brain (Supplementary Figure S9). Thus, despite the A*β* load, the paracellular barrier of the BBB was preserved in 5xFAD mice.

### 5xFAD mice exhibit no signs of fibrinogen extravasation

**Figure 5:**
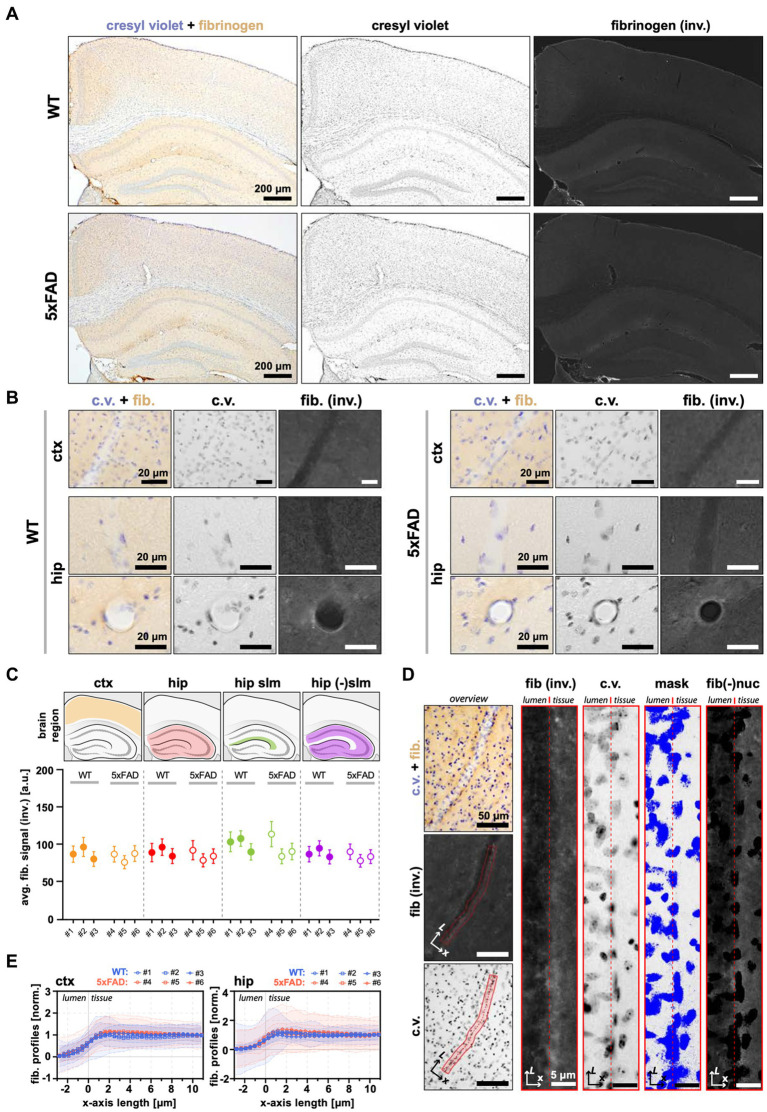
No apparent fibrinogen leakage in 5xFAD mice. **(A)** Representative bright-filed microscopy images of coronal brain sections from WT and 5xFAD mice showing the cortex and hippocampus immunostained for cresyl violet (to visualize cell nuclei) and fibrinogen. **(B)** The staining in proximity to a blood vessel. **(C)** Quantification of fibrinogen content in the brain. The average fibrinogen intensity in the cortex, hippocampus, and its subregions are similar between WT and 5xFAD. The upper panel indicates the analyzed areas with corresponding anatomical locations. The lower panel shows average signal intensities within each analyzed animal (demarked with #; *n* (WT) = 3, *n* (5xFAD) = 3). The data is presented as the average and standard deviation (STD) of the signal measured from a region-wide ROI. **(D)** The principle of fibrinogen analysis at the BBB interface. The left panels show the perpendicularly oriented vessel manually tracked at the vessel lumen/parenchyma boundary along its long axis (L). The right panels show the same selection straightened. From left: the fibrinogen channel; cresyl violet channel; threshold mask to exclude areas occupied by nuclei; and the resulting image used to calculate the fibrinogen vessel profile (see also “Methods” section). **(E)** Fibrinogen vessel profiles in the cortex and hippocampus show no trend toward the enrichment of fibrinogen at the interface between the vessel lumen and the brain tissue in 5xFAD mice. The thick lines correspond to the weighted average for each animal, with shaded areas showing accompanying STD (see Methods section). The data on the plot was normalized to the values in a range [0.0.1], where 0 is the lowest signal intensity (vessel lumen), and 1 is the background signal (the brain parenchyma, i.e., the average of the last 10 points at the x-axis). Because of the high overlap of intensity profiles, only each 10-th datapoint was plotted. All panels: (inv.) = inverted image; c.v. = cresyl violet; fib. = fibrinogen; fib(−)nuc = fibrinogen channel with excluded nuclei areas; ctx = cortex; hip = hippocampus; a.u. = arbitrary units of pixel intensity; L = vessel length axis; x = profile plot length axis; ROI = region of interest.

Extravasation of blood-borne fibrinogen is another sign of compromised BBB and a contributing factor to AD progression, observed in some patients (Ryu and McLarnon, 2009), and reported in several animal models of AD (Cortes-Canteli et al., 2012). We measured the extravasation of fibrinogen by IHC in 7 m.o. WT and 5xFAD mice (Figure 5). Given the contribution of hippocampal pathology to AD, we included the hippocampus in our analyses. Neither WT nor 5xFAD exhibited signs of fibrinogen extravasation in corresponding brain regions (Figures 5A,B). First, we measured the average signal from the brain tissue in the cortex and in the hippocampus, separating the latter into stratum lacunosum-moleculare (s.l-m) and remaining areas due to non-uniform distribution of the baseline signal, i.e., higher in the s.l-m (Figures 5A,C). Regardless of the brain region, we found no fibrinogen leakage in 5xFAD mice, as evidenced by consistent levels of the measured signal between WT and 5xFAD mice (Figure 5C). Although whole-brain analysis can provide the measure of the degree of fibrinogen leak across the BBB, it may not be sensitive for local, i.e., perivascular increase in fibrinogen content. Therefore, we next analyzed the fibrinogen intrusion at the microscale, i.e., in the areas proximal to the brain microvessels. We extracted the fibrinogen signal intensity profiles along the *>*20 μm diameter brain vessels, obtaining profile plots across the boundary regions between the vessel lumen and the neighboring brain tissue (Figure 5D). In accord with the results from wide-brain areas, we observed no trend toward a local increase in fibrinogen content, with profile plots across the BBB interface and neighboring parenchyma being similar between WT and 5xFAD mice for both cortical (3–7 vessels per animal; *n* = 3 WT, *n* = 3 5xFAD) and hippocampal microvessels (2–5 vessels per animal; *n* = 3 WT, *n* = 3 5xFAD; Figure 5E). Thus, the BBB in 5xFAD mice exhibited no increase in permeability toward blood-borne fibrinogen.

### Neurovascular coupling is not altered in 5xFAD mice

**Figure 6:**
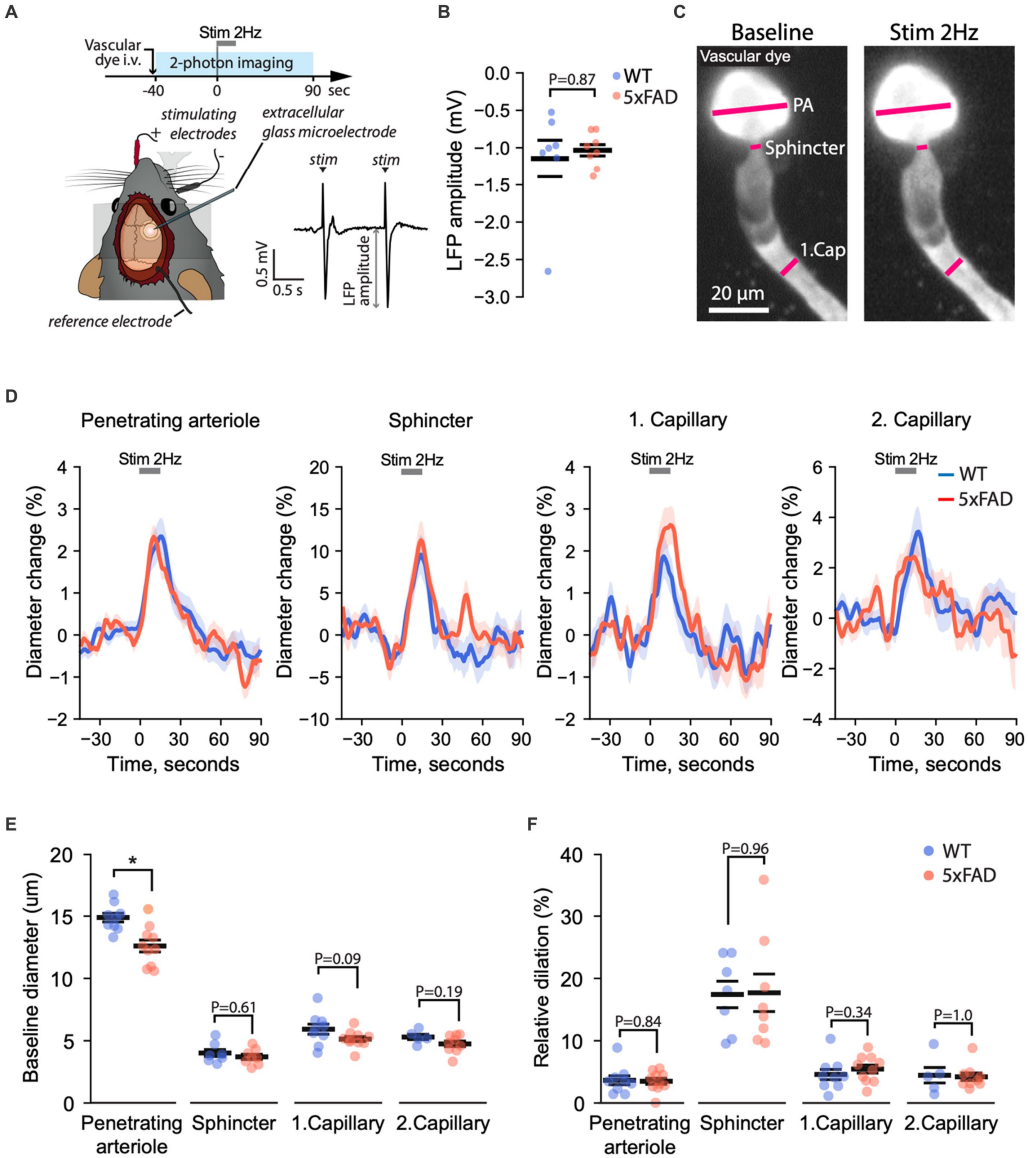
Neurovascular coupling is unaffected in 5xFAD mice. **(A)** Experiment timeline (top), a schematic of the mouse head with electrodes stimulating the whisker pad and recording the LFP (bottom left), and an example of stimulus-evoked LFP (bottom right). The amplitude of the negative deflection of the LFP (LFP amplitude) was quantified to assess brain activation. **(B)** The amplitude of local-field potentials did not differ between 5xFAD and WT mice, indicating equal cortical activation during whisker pad stimulation (Mann–Whitney U test at the significance level 0.05; *n* (WT) = 7, *n* (5xFAD) = 8). **(C)** The diameters of penetrating arteriole (PA), precapillary sphincter, and downstream capillaries were quantified simultaneously from projected z-stack images recorded over time before, during, and after stimulation. **(D)** Relative diameter change over time before, during and after stimulation in respective vessel segments in 5xFAD and WT mice. **(E)** Quantification of the resting diameter (Student’s *T*-test, * *p <* 0.05; number of data points for PA *n* (WT) = 8, *n* (5xFAD) = 8, for precapillary sphincter *n* (WT) = 5, *n* (5xFAD) = 5, for 1st-order capillary *n* (WT) = 8, *n* (5xFAD) = 8, for 2nd-order capillary *n* (WT) = 4, *n* (5xFAD) = 5). **(F)** Quantification of the peak diameter change relative to the baseline diameter [Student’s *T*-test at the significance level 0.05; the number of data points is the same as in panel **(E)**]. PA, penetrating arteriole; Sph, Sphincter; 1stCap, 1st-order capillary; 2ndCap, 2nd-order capillary. Panel **(A)** was adapted from Kucharz et al. (2021) under CC-BY 4.0 license. Images in **(C)** are maximum-intensity projections.

NVC ensures appropriate blood supply to satisfy an increase in metabolic demand in neurons following their activation (Hall et al., 2014; Cai et al., 2018; Zambach et al., 2021). Here, we assessed whether A*β* pathology affects NVC (Figure 6A). We stimulated the whisker pad and simultaneously monitored both evoked cortical LFP and vasodilation of individual microvessels in the somatosensory (i.e., barrel) cortex. To examine the synaptic input to evoke NVC responses, we compared the amplitudes of LFPs in WT and 5xFAD mice. The amplitude of the LFPs was the same in WT and 5xFAD mice (Median (25-th, 75-th percentiles): 1.03 (0.80, 1.24) vs. 1.02 (0.89, 1.22), *p* = 0.82, *n* (WT) = 8, *n* (5xFAD) = 10, two-tailed Mann–Whitney U test), confirming no change in 5xFAD mice (Figure 6B). This indicated equal synaptic input to evoke NVC in WT and 5xFAD mice. In parallel, we monitored NVC vasodilation by recording a time-series of Z-stack images (i.e., hyperstack) capturing penetrating arteriole, precapillary sphincter, 1st-order capillary, and 2nd-order capillaries (Figure 6C; Supplementary Video S7). We quantified the baseline diameter and the relative dilation during stimulation by placing rectangle ROI on vessel segments (Cai et al., 2018; Figure 6D). We found that the baseline diameter of the penetrating arterioles was significantly larger in WT than in 5xFAD mice (14.8 ± 0.41 µm vs. 12.8 ± 0.56 µm, *p* = 0.018, *n* (WT) = 9, *n* (5xFAD) = 10, two-tailed Student-*t* test; Figure 6E). However, the relative dilation was the same in 5xFAD and WT mice across all corresponding vascular segments (Figure 6F). Similar to BBB experiments, we assessed whether the measured variables depended on the age of animals, and we observed no significant changes in NVC and the baseline diameter with the mouse age (Supplementary Figures S6C, S10). Thus, our results show that NVC was preserved in 5xFAD mice.

### Capillary pericyte density remains unchanged in 5xFAD mice

Pericytes are essential components of the neurovascular unit, regulating the NVC responses, structural integrity and transport across the BBB (Armulik et al., 2010; Hall et al., 2014). Loss of pericytes and their coverage is an important predictor of vascular cognitive impairment and dementia (Uemura et al., 2020), and accelerates AD-like neurodegeneration in mice (Sagare et al., 2013). Therefore, we next quantified the density of capillary pericytes in 5xFAD mice using immunohistochemistry. The tissue was immunostained for a cluster of differentiation 31 (CD31; = platelet endothelial cell adhesion molecule; PECAM-1) and platelet-derived growth factor receptor beta (PDGFR-*β*), to visualize both microvasculature and accompanying pericytes, respectively (Supplementary Figure S11A). The capillary pericytes exhibited their typical morphology of a “bump on a log” with extensive projections along the microvasculature (Supplementary Figures S11A,B). Noteworthy, IHC in 5xFAD mice also revealed the clear presence of A*β* plaques, often in proximity to capillaries (Supplementary Figure S11C) and of similar morphology to aggregates observed using 2 PM *in vivo* (Figure 1; Supplementary Videos S1, S2). Despite the presence of A*β* plaques, we found no evident reduction of the capillary pericyte density in 5xFAD mice (*n* = 3 mice/group; n Z-stack images = 10/animal; Supplementary Figure S11D). Thus, the lack of NVC and BBB dysfunction in 5xFAD animals was accompanied by preserved number of pericytes on capillaries.

Overall, the results of our study show that the major functions of the neurovascular unit were preserved in adult female 5xFAD mice despite the widespread presence of A*β* plaques in the brain.

## Discussion

A common assumption is that vascular dysfunction is inherent to A*β* deposition, but recent evidence, including the current study, suggests that this view may be oversimplified (Supplementary Table S3). Here we report that in 5xFAD female mice, an aggressive A*β* model of AD, major functions of the neurovascular unit, namely BBB and NVC, are preserved despite the extensive A*β* plaque burden.

One of the major aspects of BBB dysfunction is an increase in AMT. In a healthy brain, AMT is suppressed by homeostatic mechanisms (Andreone et al., 2017; Ayloo and Gu, 2019; Mathiesen Janiurek et al., 2019). However, in aging, neuroinflammation, and after stroke, AMT is disinhibited, allowing a surge of macromolecules into the brain which, in turn, can cause and facilitate neurodegeneration (Knowland et al., 2014; Sadeghian et al., 2018; Yang et al., 2020; Erickson and Banks, 2022; Zhu et al., 2022). Whether AMT is increased in AD-like pathology remained hitherto unaddressed.

Recent advances allowed monitoring of AMT at unprecedented details *in vivo*, i.e., at different categories of cerebral microvessels and at the level of single transport vesicles in the living brain (Mathiesen Janiurek et al., 2019; Kucharz et al., 2021; Thomsen et al., 2022). Here, using 2 PM, we found that AMT was unchanged in 5xFAD mice compared to the WT, regardless of the vessel type. Even penetrating arterioles which exhibit the highest susceptibility to AMT increase (Bell et al., 2012; Mathiesen Janiurek et al., 2019), and are a preferred locus of amyloid-beta deposition along the microvascular tree (Tarasoff-Conway et al., 2015), had no signs of altered AMT. These results might be unexpected, given that the AMT increase is generally considered the most sensitive and early indicator of the BBB pathology that precedes alterations in the brain endothelial cells ultrastructure (Knowland et al., 2014; Mathiesen Janiurek et al., 2019). However, comparisons to other BBB studies should be made with caution as the current knowledge about the onset and progression of AMT dysfunction is based on studies of acute brain pathology (Knowland et al., 2014; Sadeghian et al., 2018; Zhu et al., 2022) or aging (Yang et al., 2020), with no corresponding longitudinal assessments in AD. Although we cannot exclude the presence of compensatory mechanisms buffering the AMT dysfunction in AD, so far, such mechanisms have only been reported regarding neuronal function, but with no insights into transcytosis (Elman et al., 2014).

Another aspect of compromised BBB is increased paracellular permeability, typically attributed to abnormal junctional complexes between adjoining brain endothelial cells (Sweeney et al., 2018b). At the functional level, the increased leakage across the BBB has been found in 5xFAD mice at 9–10 m.o. using NaFluo fluorescence tracer (Park et al., 2017) and at *>*18 m.o. using gadolinium as a contrast agent (Montagne et al., 2021). This may contrast with findings in 5xFAD at the intermediate age (6 m.o.), where the BBB permeability was unchanged toward NaFluo (Marottoli et al., 2017; Ries et al., 2021). Other studies also used Evans Blue to address changes in paracellular permeability, a common method, but neither specific toward paracellular leakage nor AMT (Saunders et al., 2015). Nonetheless, the Evans Blue extravasation has been reported in young (3–5 m.o.) mice (Liu et al., 2020; Ries et al., 2021). Here, we used NaFluo, as a tracer for assessing BBB paracellular permeability (Kutuzov et al., 2018; Mathiesen Janiurek et al., 2019). In addition, we also measured the extravasation of fibrinogen, an endogenous marker of BBB dysfunction. We report that the BBB was functionally intact in 7–11-m.o. 5xFAD mice, with no signs of increased leakage toward both NaFluo and fibrinogen. This aligns with previous findings questioning the view that a widespread A*β* deposition in the cerebral cortex is sufficient to induce a rise in paracellular permeability (Bien-Ly et al., 2015; Marottoli et al., 2017; Sasaguri et al., 2017; Ries et al., 2021). The BBB in the 5xFAD mice may become dysfunctional at an early age (Ries et al., 2021), regain the barrier properties at an intermediate age (Ries et al., 2021, and this study), and later lose its function again at an advanced age (Montagne et al., 2021). Although the plausibility of this two-stage dysfunction of the BBB in the 5xFAD model may be apparent from the literature, a future longitudinal study assessing the BBB will be needed to test this hypothesis. Even so, it becomes apparent that the conjoined presence of BBB deficits and A*β* plaques is not always the case in the 5xFAD preclinical model of AD, which is consistent with clinical data (Profaci et al., 2020; Jansen et al., 2022) [however, see also (Selkoe and Hardy, 2016)].

Dysregulation of CBF accompanies AD pathology as indicated by reduced baseline CBF (Bracko et al., 2021), attenuation of autoregulation (Niwa et al., 2002) and impairment of NVC (Niwa et al., 2000; Iadecola, 2017; Mughal et al., 2021). Soluble and aggregated A*β* can contribute to NVC dysfunction, as was found in both young and aged AD mice (Niwa et al., 2000; Park et al., 2014; Kimbrough et al., 2015; Shabir et al., 2022). For example, 3–4-m.o. Tg2576, Tg-SwDI, and Duch/Iowa mice (Niwa et al., 2000; Takano et al., 2007; Park et al., 2014) as well as 30-m.o. J20 mice (Kimbrough et al., 2015) demonstrated a significant reduction of CBF response upon sensory stimulation. These studies recorded CBF responses using either laser-doppler flowmetry (LDF) or optical imaging spectroscopy (OIS). However, these methods cannot discern the contribution of individual vessels because of the limited spatial resolution. Although few studies assessed the reactivity of individual capillaries in brain slices (Nortley et al., 2019) and isolated arterioles (Dietrich et al., 2010), no study examined NVC at individual vessels in the presence of A*β in vivo*.

Here, we examined NVC at the level of arterioles, capillaries, and precapillary sphincters in the living brain. Our imaging data shows that the baseline diameter of penetrating arterioles was smaller in 5xFAD mice than in WT, which may explain the reduction of the CBF baseline observed in 7-m.o. and older 5xFAD mice (Igarashi et al., 2020; Shabir et al., 2022). Noteworthy, in the APP^NL-G-F^ model of AD, A*β* did not induce constriction of arterioles but only capillaries (Nortley et al., 2019). It is feasible that the distinct effect of A*β* on the microvessels in APP^NL-G-F^ and 5xFAD stems from the differences in expression of A*β*, being lower in APP^NL-G-F^, which translates to fewer plaques and their smaller size compared to 5xFAD mice (Sasaguri et al., 2017; Locci et al., 2021), but more evidence is needed to test this hypothesis.

Regarding CBF responses to whisker stimulation, NVC was previously found to be reduced in 5xFAD mice at 7 and 12–13 m.o. (Lazic et al., 2019; Mughal et al., 2021). Here, we found no difference in NVC between 5xFAD and WT at 7–11 m.o. The divergence between Lazic et al. (2019) and Mughal et al. (2021), and our findings may be related to differences in methodological approaches (e.g., anesthesia, stimulation paradigms) as well as the age of mice. The latter can affect the amount of soluble A*β*_1–40_ and A*β*_1–42_, both present in 5xFAD mice (Oakley et al., 2006; Maarouf et al., 2013; Bhattacharya et al., 2014; Abe et al., 2020). Mechanistically, it is A*β*_1–40_ and tau, rather than A*β*_1–42_, that dysregulate NVC (Niwa et al., 2000; Park et al., 2020), but 5xFAD mice express predominantly A*β*_1–42_ and no tau (Oakley et al., 2006; Sasaguri et al., 2017; Abe et al., 2020; Forner et al., 2021). It is plausible that at the studied age, the amount of A*β*_1–40_ was too small in 5xFAD to dysregulate NVC. Nevertheless, our current study suggests that A*β* alone might not suffice to dysregulate NVC, which is in accord with previous studies reporting normal neurovascular coupling in other mouse models of AD (Shin et al., 2007; Duncombe et al., 2017). In addition, previous studies reported normal EPSCs in the cortex of 2–4-m.o. 5xFAD (B6/SJL background) and normal behavioral performance and EEG in 2–12-m.o. 5xFAD (C57BL/6J background) mice (Andersen et al., 2021; Oblak et al., 2021).

Lastly, we show that the density of capillary pericytes was the same in WT and 5xFAD mice. Although the pericyte coverage reportedly decreases in older 5xFAD mice (Montagne et al., 2021), our findings agree with reports in 5xFAD mice of the same age as used in our study (Damisah et al., 2017). While we cannot exclude that, despite the preserved pericyte density, there were changes in pericyte coverage or intracellular signaling, such changes were insufficient to impair both BBB and NVC in 5xFAD mice.

An important aspect of our study is the choice of animal model. We used female 7–11-m.o. 5xFAD mice on a mixed B6/SJL background to characterize the BBB and NVC under the condition of extensive A*β* burden. 5xFAD mice accumulate A*β* from early age, faster than other models (Oakley et al., 2006; Jawhar et al., 2012) and also to a greater extent compared to, e.g., APP/PS1 and APP^NL-G-F^ mice (Sasaguri et al., 2017; Locci et al., 2021). From 7 months of age, 5xFAD mice already exhibit a considerable amount of A*β* and neurological deficits (Jawhar et al., 2012; Eimer and Vassar, 2013; Sil et al., 2022). We used female mice because they express more APP, accumulate more A*β*, and develop stronger neuroinflammation than males (Oakley et al., 2006; Maarouf et al., 2013; Forner et al., 2021; Sil et al., 2022). The mixed B6/SJL background further facilitates A*β* production (Neuner et al., 2019). Importantly, Aβ deposition is smaller in superficial cortical layers than in deeper layers in 5xFAD mice (Oblak et al., 2021), which may influence *in vivo* findings reported here. However, estimates of Aβ plaque densities differ between studies (Whitesell et al., 2019) and should be considered with caution. Although the somatosensory stimulus follows the thalamic route, which first activates deeper layers of the cortex, and then layers II/III, it is unclear whether preserved NVC in cortical depths up to 250 μm guarantee intact NVC in deeper cortical layers. In addition, the Trem2S148E allele in B6/SJL background may affect microglia and, hence, the NVC (Yang et al., 2021; Császár et al., 2022); however, the exact effects of Trem2S148E remain unstudied. Also, NVC and BBB may be affected by the age of the animal (Park et al., 2014; Sweeney et al., 2018a; Yang et al., 2020; Banks et al., 2021; Cai et al., 2023). Although our data suggest that NVC and BBB are unaffected by mouse age between 7–11 months, longitudinal studies that cover the whole lifespan are favorable to monitor the animals throughout the onset of signs and development of the disease. Finally, cerebral amyloid angiopathy (CAA) was not found in parenchymal vessels in 5xFAD mice (Giannoni et al., 2016; Marazuela et al., 2022). Because CAA can affect vascular function (Magaki et al., 2018), lack of CAA in 5xFAD is another possible determinant of our findings.

Here, we characterized BBB and NVC using 2 PM in cortical depth up to 250 μm, and supplemented our findings with IHC analysis of BBB leakage to fibrinogen in the cortex and hippocampus. We assessed leakage of fibrinogen, which is readily detectable with bright-field microscopy (Ryu and McLarnon, 2009), but was absent herein. Fluorescent labeling and confocal microscopy may be suitable for analyses of possibly minimal presence of fibrinogen in the 5xFAD mice brains (Cortes-Canteli et al., 2010). Compared to other *in vivo* techniques (DC-MRI, LDF, LSI), 2 PM has a superior spatial resolution. This allows 2 PM to investigate the properties of microcirculation on the level of single vessels and spatially separate the BBB tracer signal in the brain parenchyma from its signal in the vessel lumen, which is crucial for reliable quantification of the BBB permeability (Kucharz et al., 2022). Nevertheless, 2 PM has limitations. First, although AMT and NVC were characterized at individual brain vessels, the BBB permeability was based on the signal from parenchyma that integrates contributions of all microvessels. Second, AMT was quantified by counting BSA-containing vesicles at the vascular endothelium. Counting vesicles is an indirect estimate of AMT used previously by both 2 PM (Mathiesen Janiurek et al., 2019) and transmission electron microscopy (Ben-Zvi et al., 2014; Knowland et al., 2014; Andreone et al., 2017; Yang et al., 2020; Zhu et al., 2022). In contrast, direct quantification of transcytosis requires simultaneous tracking of an individual tracer molecule at the BBB and subsequently in the brain parenchyma *in vivo*, a project of utmost complexity that, to our knowledge, still awaits its implementation (Kucharz et al., 2022).

In summary, we found that A*β* does not warrant BBB and NVC dysfunction. Our results call for careful interpretation and avoiding generalizations of the findings obtained across distinct preclinical models of AD. This concerns not only BBB and NVC but may extend to a broader range of microvascular functions and other aspects of AD pathology.

## Data availability statement

The raw data supporting the conclusions of this article will be made available by the authors, without undue reservation.

## Ethics statement

The animal study was reviewed and approved by The Danish National Committee on Health Research Ethics.

## Author contributions

KK, ML, CC, BA, MB, RSK and OZ contributed to conception and design of the study. OZ performed the *in vivo* experiments and gathered the data. RSK and KK performed the *ex vivo* experiments and gathered the data. OZ, CH, CC, AL, and KK analyzed the data. OZ, KK, ML wrote the first draft of the manuscript. AL, CC, and RSK wrote sections of the manuscript. All authors contributed to manuscript revision, read, and approved the submitted version.

## Funding

This study was supported by the Lundbeck Foundation (#R392-2018-2266 and #R345-2020-1440), the Danish Medical Research Council (#1030-00374A), the NOVO Nordisk Foundation (#117272), a Nordea Foundation grant to the Center for Healthy Aging (#114995), and the Alzheimer-forskningsfonden (#122137). The grants covered researchers’ salaries and the running cost of the experiments.

## Conflict of interest

The authors declare that the research was conducted in the absence of any commercial or financial relationships that could be construed as a potential conflict of interest.

## Publisher’s note

All claims expressed in this article are solely those of the authors and do not necessarily represent those of their affiliated organizations, or those of the publisher, the editors and the reviewers. Any product that may be evaluated in this article, or claim that may be made by its manufacturer, is not guaranteed or endorsed by the publisher.

## References

[ref1] Abe Y. Ikegawa N. Yoshida K. Muramatsu K. Hattori S. Kawai K.. (2020). Behavioral and electrophysiological evidence for a neuroprotective role of aquaporin-4 in the 5xFAD transgenic mice model. Acta Neuropathol. Commun. 8:67. doi: 10.1186/s40478-020-00936-3, PMID: 32398151 PMC7218576

[ref2] Andersen J. V. Skotte N. H. Christensen S. K. Polli F. S. Shabani M. Markussen K. H.. (2021). Hippocampal disruptions of synaptic and astrocyte metabolism are primary events of early amyloid pathology in the 5xFAD mouse model of Alzheimer’s disease. Cell Death Dis. 12:954. doi: 10.1038/s41419-021-04237-y, PMID: 34657143 PMC8520528

[ref3] Andreone B. J. Chow B. W. Tata A. Lacoste B. Ben-Zvi A. Bullock K.. (2017). Blood Brain barrier permeability is regulated by lipid transport-dependent suppression of Caveolae-mediated transcytosis. Neuron 94, 581–594.e5. doi: 10.1016/j.neuron.2017.03.043, PMID: 28416077 PMC5474951

[ref4] Armulik A. Genové G. Mäe M. Nisancioglu M. H. Wallgard E. Niaudet C.. (2010). Pericytes regulate the blood-brain barrier. Nature 468, 557–561. doi: 10.1038/nature09522 20944627

[ref5] Asgari Taghanaki S. Abhishek K. Cohen J. P. Cohen-Adad J. Hamarneh G. (2021). Deep semantic segmentation of natural and medical images: a review. Artif. Intell. Rev. 54, 137–178. doi: 10.1007/s10462-020-09854-1

[ref6] Ayloo S. Gu C. (2019). Transcytosis at the blood–brain barrier. Curr. Opin. Neurobiol. 57, 32–38. doi: 10.1016/j.conb.2018.12.014, PMID: 30708291 PMC6629499

[ref7] Banks W. A. Erickson M. A. (2010). The blood-brain barrier and immune function and dysfunction. Neurobiol. Dis. 37, 26–32. doi: 10.1016/j.nbd.2009.07.031 19664708

[ref8] Banks W. A. Reed M. J. Logsdon A. F. Rhea E. M. Erickson M. A. (2021). Healthy aging and the blood–brain barrier. Nature Aging 1, 243–254. doi: 10.1038/s43587-021-00043-5, PMID: 34368785 PMC8340949

[ref9] Bell R. D. Winkler E. A. Singh I. Sagare A. P. Deane R. Wu Z.. (2012). Apolipoprotein e controls cerebrovascular integrity via cyclophilin a. Nature 485, 512–516. doi: 10.1038/nature11087, PMID: 22622580 PMC4047116

[ref10] Ben-Zvi A. Lacoste B. Kur E. Andreone B. J. Mayshar Y. Yan H.. (2014). Mfsd2a is critical for the formation and function of the blood-brain barrier. Nature 509, 507–511. doi: 10.1038/nature13324, PMID: 24828040 PMC4134871

[ref11] Bhattacharya S. Haertel C. Maelicke A. Montag D. (2014). Galantamine slows down plaque formation and behavioral decline in the 5XFAD mouse model of Alzheimer’s disease. PLoS One 9:e89454. doi: 10.1371/journal.pone.0089454, PMID: 24586789 PMC3931790

[ref12] Bien-Ly N. Boswell C. A. Devoss J. Van Der Brug M. Watts Correspondence R. J. Jeet S.. (2015). Lack of widespread BBB disruption in Alzheimer’s disease models: focus on therapeutic antibodies. Neuron 88, 289–297. doi: 10.1016/j.neuron.2015.09.036, PMID: 26494278

[ref13] Bracko O. Cruz Hernandez J. C. Park L. Nishimura N. Schaffer C. B. (2021). Causes and´ consequences of baseline cerebral blood flow reductions in Alzheimer’s disease. J. Cereb. Blood Flow Metab. 41, 1501–1516. doi: 10.1177/0271678X20982383, PMID: 33444096 PMC8221770

[ref14] Cai C. Fordsmann J. C. Jensen S. H. Gesslein B. Lønstrup M. Hald B. O.. (2018). Stimulation induced increases in cerebral blood flow and local capillary vasoconstriction depend on conducted vascular responses. Proc. Natl. Acad. Sci. U. S. A. 115, E5796–E5804. doi: 10.1073/pnas.1707702115, PMID: 29866853 PMC6016812

[ref15] Cai C. Zambach S. A. Grubb S. Tao L. He C. Lind B. L.. (2023). Impaired dynamics of precapillary sphincters and pericytes at first-order capillaries predict reduced neurovascular function in the aging mouse brain. Nature Aging 3, 173–184. doi: 10.1038/s43587-022-00354-1 37118115 PMC11081516

[ref16] Chow B. W. Nunez V. Kaplan L. Granger A. J. Bistrong K. Zucker H. L.. (2020). Caveolae in CNS arterioles mediate neurovascular coupling. Nature 579, 106–110. doi: 10.1038/s41586-020-2026-1, PMID: 32076269 PMC7060132

[ref17] Cortes-Canteli M. Paul J. Norris E. H. Bronstein R. Ahn H. J. Zamolodchikov D.. (2010). Fibrinogen and Beta-amyloid association alters thrombosis and fibrinolysis: a possible contributing factor to Alzheimer’s disease. Neuron 66, 695–709. doi: 10.1016/j.neuron.2010.05.014, PMID: 20547128 PMC2895773

[ref18] Cortes-Canteli M. Zamolodchikov D. Ahn H. J. Strickland S. Norris E. H. (2012). Fibrinogen and altered hemostasis in Alzheimer’s disease. J. Alzheimers Dis. 32, 599–608. doi: 10.3233/JAD-2012-120820, PMID: 22869464 PMC3683985

[ref19] Császár E. Lénárt N. Cserép C. Kórnyei Z. Fekete R. Pösfai B.. (2022). Microglia modulate blood flow, neurovascular coupling, and hypoperfusion via purinergic actions. J. Exp. Med. 219:e20211071. doi: 10.1084/jem.20211071, PMID: 35201268 PMC8932534

[ref20] Damisah E. C. Hill R. A. Tong L. Murray K. N. Grutzendler J. (2017). A fluoro-nissl dye identifies pericytes as distinct vascular mural cells during *in vivo* brain imaging. Nat. Neurosci. 20, 1023–1032. doi: 10.1038/nn.4564, PMID: 28504673 PMC5550770

[ref21] Dice L. R. (1945). Measures of the amount of ecologic association between species. Ecology 26, 297–302. doi: 10.2307/1932409

[ref22] Dietrich H. H. Xiang C. Han B. H. Zipfel G. J. Holtzman D. M. (2010). Soluble amyloid-beta, effect on cerebral arteriolar regulation and vascular cells. Mol. Neurodegener. 5:15. doi: 10.1186/1750-1326-5-15, PMID: 20388225 PMC2873254

[ref23] Duncombe J. Lennen R. J. Jansen M. A. Marshall I. Wardlaw J. M. Horsburgh K. (2017). Ageing causes prominent neurovascular dysfunction associated with loss of astrocytic contacts and gliosis. Neuropathol. Appl. Neurobiol. 43, 477–491. doi: 10.1111/nan.12375, PMID: 28039950

[ref24] Eimer W. A. Vassar R. (2013). Neuron loss in the 5XFAD mouse model of Alzheimer’s disease correlates with intraneuronal A*β*42 accumulation and caspase-3 activation. Mol. Neurodegener. 8:2. doi: 10.1186/1750-1326-8-2, PMID: 23316765 PMC3552866

[ref25] Elman J. A. Oh H. Madison C. M. Baker S. L. Vogel J. W. Marks S. M.. (2014). Neural compensation in older people with brain amyloid-*β* deposition. Nat. Neurosci. 17, 1316–1318. doi: 10.1038/nn.3806, PMID: 25217827 PMC4177011

[ref26] Erickson M. A. Banks W. A. (2022). Transcellular routes of blood-brain barrier disruption. Exp. Biol. Med. 247, 788–796. doi: 10.1177/15353702221080745, PMID: 35243912 PMC9134765

[ref27] Forner S. Kawauchi S. Balderrama-Gutierrez G. Kramar E. A. Matheos D. P. Phan J.. (2021). Systematic phenotyping and characterization of the 5xFAD mouse model of Alzheimer’s disease. Sci. Data 8:270. doi: 10.1038/s41597-021-01054-y, PMID: 34654824 PMC8519958

[ref28] Gao Y. Liu Q. Xu L. Zheng N. He X. Xu F. (2019). Imaging and spectral characteristics of amyloid plaque autofluorescence in brain slices from the APP/PS1 mouse model of Alzheimer’s disease. Neurosci. Bull. 35, 1126–1137. doi: 10.1007/s12264-019-00393-6, PMID: 31127445 PMC6864001

[ref29] Giannoni P. Arango-Lievano M. Neves I. D. Rousset M. C. Baranger K. Rivera S.. (2016). Cerebrovascular pathology during the progression of experimental Alzheimer’s disease. Neurobiol. Dis. 88, 107–117. doi: 10.1016/j.nbd.2016.01.001, PMID: 26774030

[ref30] Grubb S. Cai C. Hald B. O. Khennouf L. Murmu R. P. Jensen A. G. K.. (2020). Precapillary sphincters maintain perfusion in the cerebral cortex. Nat. Commun. 11:11. doi: 10.1038/s41467-020-14330-z 31959752 PMC6971292

[ref31] Gustafsson S. Lindstrom V. Ingelsson M. Hammarlund-Udenaes M. Syvänen S. (2018). Intact blood-brain barrier transport of small molecular drugs in animal models of amyloid beta and alpha synuclein pathology. Neuropharmacology 128, 482–491. doi: 10.1016/j.neuropharm.2017.08.002, PMID: 28797721

[ref32] Hall C. N. Reynell C. Gesslein B. Hamilton N. B. Mishra A. Sutherland B. A.. (2014). Capillary pericytes regulate cerebral blood flow in health and disease. Nature. [Dataset] 508, 55–60. doi: 10.1038/nature13165, PMID: 24670647 PMC3976267

[ref33] Hartz A. M. S. Bauer B. Soldner E. L. B. Wolf A. Boy S. Backhaus R.. (2012). Amyloid-*β* contributes to blood-brain barrier leakage in transgenic human amyloid precursor protein mice and in humans with cerebral amyloid angiopathy. Stroke 43, 514–523. doi: 10.1161/STROKEAHA.111.627562, PMID: 22116809 PMC5761312

[ref34] Iadecola C. (2017). The neurovascular unit coming of age: a journey through neurovascular coupling in health and disease. Neuron 96, 17–42. doi: 10.1016/j.neuron.2017.07.030, PMID: 28957666 PMC5657612

[ref35] Igarashi H. Ueki S. Kitaura H. Kera T. Ohno K. Ohkubo M.. (2020). Longitudinal GluCEST MRI changes and cerebral blood flow in 5xFAD mice. Contrast Media Mol. Imaging 2020:8831936. doi: 10.1155/2020/8831936, PMID: 33304204 PMC7714610

[ref36] Jansen W. J. Janssen O. Tijms B. M. Vos S. J. B. Ossenkoppele R. Visser P. J.. (2022). Prevalence estimates of amyloid abnormality across the Alzheimer disease clinical spectrum. JAMA Neurol. 79, 228–243. doi: 10.1001/jamaneurol.2021.5216, PMID: 35099509 PMC12138908

[ref37] Jawhar S. Trawicka A. Jenneckens C. Bayer T. A. Wirths O. (2012). Motor deficits, neuron loss, and reduced anxiety coinciding with axonal degeneration and intraneuronal A*β* aggregation in the 5XFAD mouse model of Alzheimer’s disease. Neurobiol. Aging 33, 196.e29–196.e40. doi: 10.1016/j.neurobiolaging.2010.05.027, PMID: 20619937

[ref38] Jessen S. B. Brazhe A. Lind B. L. Mathiesen C. Thomsen K. Jensen K.. (2015). GABAA receptor-mediated bidirectional control of synaptic activity, intracellular ca2+, cerebral blood flow, and oxygen consumption in mouse somatosensory cortex *in vivo*. Cereb. Cortex 25, 2594–2609. doi: 10.1093/cercor/bhu058, PMID: 24692513

[ref39] Kimbrough I. F. Robel S. Roberson E. D. Sontheimer H. (2015). Vascular amyloidosis impairs the gliovascular unit in a mouse model of Alzheimer’s disease. Brain 138, 3716–3733. doi: 10.1093/brain/awv327, PMID: 26598495 PMC5006220

[ref40] Klohs J. Politano I. W. Deistung A. Grandjean J. Drewek A. Dominietto M.. (2013). Longitudinal assessment of amyloid pathology in transgenic ArcA*β* mice using multi-parametric magnetic resonance imaging. PLoS One 8:e66097. doi: 10.1371/journal.pone.0066097, PMID: 23840405 PMC3686820

[ref41] Knowland D. Arac A. Sekiguchi K. J. Hsu M. Lutz S. E. Perrino J.. (2014). Stepwise recruitment of transcellular and paracellular pathways underlies blood-brain barrier breakdown in stroke. Neuron 82, 603–617. doi: 10.1016/j.neuron.2014.03.003, PMID: 24746419 PMC4016169

[ref42] Kook S. Y. Hong H. S. Moon M. Ha C. M. Chang S. Mook-Jung I. (2012). A*β* 1-42-rage interaction disrupts tight junctions of the blood-brain barrier via ca 2+−calcineurin signaling. J. Neurosci. 32, 8845–8854. doi: 10.1523/JNEUROSCI.6102-11.2012, PMID: 22745485 PMC6622350

[ref43] Kucharz K. Kristensen K. Johnsen K. B. Lund M. A. Lønstrup M. Moos T.. (2021). Postcapillary venules are the key locus for transcytosis-mediated brain delivery of therapeutic nanoparticles. Nat. Commun. 12:4121. doi: 10.1038/s41467-021-24323-1 34226541 PMC8257611

[ref44] Kucharz K. Kutuzov N. Zhukov O. Mathiesen Janiurek M. Lauritzen M. (2022). Shedding light on the Blood–Brain barrier transport with Two-Photon microscopy *in vivo*. Pharm. Res. 39, 1457–1468. doi: 10.1007/s11095-022-03266-2 35578062

[ref45] Kucharz K. Lauritzen M. (2018). CaMKII-dependent endoplasmic reticulum fission by whisker stimulation and during cortical spreading depolarization. Brain 141, 1049–1062. doi: 10.1093/brain/awy036, PMID: 29538620

[ref46] Kutuzov N. Flyvbjerg H. Lauritzen M. (2018). Contributions of the glycocalyx, endothelium, and extravascular compartment to the blood–brain barrier. Proc. Natl. Acad. Sci. U. S. A. 115, E9429–E9438. doi: 10.1073/pnas.1802155115, PMID: 30217895 PMC6176561

[ref47] Kwan A. C. Duff K. Gouras G. K. Webb W. W. (2009). Optical visualization of Alzheimer’s pathology via multiphoton-excited intrinsic fluorescence and second harmonic generation. Opt. Express 17, 3679–3689. doi: 10.1364/oe.17.003679, PMID: 19259208 PMC2977950

[ref48] Lazic D. Sagare A. P. Nikolakopoulou A. M. Griffin J. H. Vassar R. Zlokovic B. V. (2019). 3K3A-activated protein C blocks amyloidogenic BACE1 pathway and improves functional outcome in mice. J. Exp. Med. 216, 279–293. doi: 10.1084/jem.20181035, PMID: 30647119 PMC6363429

[ref49] Liu Y. Huber C. C. Wang H. (2020). Disrupted blood-brain barrier in 5×FAD mouse model of Alzheimer’s disease can be mimicked and repaired *in vitro* with neural stem cell-derived exosomes. Biochem. Biophys. Res. Commun. 525, 192–196. doi: 10.1016/j.bbrc.2020.02.074, PMID: 32081424

[ref50] Locci A. Orellana H. Rodriguez G. Gottliebson M. McClarty B. Dominguez S.. (2021). Comparison of memory, affective behavior, and neuropathology in APPNLGF knock-in mice to 5xFAD and APP/PS1 mice. Behav. Brain Res. 404:113192. doi: 10.1016/j.bbr.2021.113192, PMID: 33607163 PMC7980131

[ref51] Maarouf C. L. Kokjohn T. A. Whiteside C. M. Macias M. P. Kalback W. M. Sabbagh M. N.. (2013). Molecular differences and similarities between Alzheimer’s disease and the 5XFAD transgenic mouse model of amyloidosis. *Biochem*. *Insights* 6, 1–10. doi: 10.4137/BCI.S13025, PMID: 25210460 PMC4154482

[ref02] Magaki S. Tang Z. Tung S. Williams C. K. Lo D. Yong W. H.. (2018). The effects of cerebral amyloid angiopathy on integrity of the blood-brain barrier. Neurobiol. Aging. 70, 70–77. doi: 10.1016/j.neurobiolaging.2018.06.004 30007166 PMC6146962

[ref01] Marazuela P. Paez-Montserrat B. Bonaterra-Pastra A. Solé M. Hernández-Guillamon M. (2022). Impact of Cerebral Amyloid Angiopathy in Two Transgenic Mouse Models of Cerebral β-Amyloidosis: A Neuropathological Study. Int. J. Mol. Sci. 23:4972. doi: 10.3390/ijms23094972 35563362 PMC9103818

[ref52] Marchiando A. M. Shen L. Graham W. V. Weber C. R. Schwarz B. T. Austin J. R. 2nd. (2010). Caveolin-1-dependent occludin endocytosis is required for TNF-induced tight junction regulation *in vivo*. J. Cell Biol. 189, 111–126. doi: 10.1083/jcb.200902153, PMID: 20351069 PMC2854371

[ref53] Marottoli F. M. Katsumata Y. Koster K. P. Thomas R. Fardo D. W. Tai L. M. (2017). Peripheral inflammation, apolipoprotein e4, and amyloid-*β* interact to induce cognitive and cerebrovascular dysfunction. ASN Neuro 9:175909141771920. doi: 10.1177/1759091417719201 PMC5521356 28707482

[ref54] Mathiesen Janiurek M. Soylu-Kucharz R. Christoffersen C. Kucharz K. Lauritzen M. (2019). Apolipoprotein m-bound sphingosine-1-phosphate regulates blood-brain barrier paracellular permeability and transcytosis. elife 8, 1–22. doi: 10.7554/eLife.49405 PMC6877292 31763978

[ref55] Montagne A. Nikolakopoulou A. M. Huuskonen M. T. Sagare A. P. Lawson E. J. Lazic D.. (2021). APOE4 accelerates advanced-stage vascular and neurodegenerative disorder in old Alzheimer’s mice via cyclophilin a independently of amyloid-*β*. Nature Aging 1, 506–520. doi: 10.1038/s43587-021-00073-z, PMID: 35291561 PMC8920485

[ref56] Mughal A. Harraz O. F. Gonzales A. L. Hill-Eubanks D. Nelson M. T. (2021). PIP2 improves cerebral blood flow in a mouse model of Alzheimer’s disease. Function 2:zqab010. doi: 10.1093/function/zqab010, PMID: 33763649 PMC7955025

[ref57] Nation D. A. Sweeney M. D. Montagne A. Sagare A. P. D’Orazio L. M. Pachicano M.. (2019). Blood–brain barrier breakdown is an early biomarker of human cognitive dysfunction. Nat. Med. 25, 270–276. doi: 10.1038/s41591-018-0297-y, PMID: 30643288 PMC6367058

[ref58] Nehra G. Bauer B. Hartz A. M. S. (2022). Blood-brain barrier leakage in Alzheimer’s disease: from discovery to clinical relevance. Pharmacol. Ther. 234:108119. doi: 10.1016/j.pharmthera.2022.108119, PMID: 35108575 PMC9107516

[ref59] Neuner S. M. Heuer S. E. Huentelman M. J. O’Connell K. M. S. Kaczorowski C. C. (2019). Harnessing genetic complexity to enhance translatability of Alzheimer’s disease mouse models: a path toward precision medicine. Neuron 101, 399–411.e5. doi: 10.1016/j.neuron.2018.11.040, PMID: 30595332 PMC6886697

[ref60] Niwa K. Kazama K. Younkin L. Younkin S. G. Carlson G. A. Iadecola C. (2002). Cerebrovascular autoregulation is profoundly impaired in mice overexpressing amyloid precursor protein. Am. J. Physiol. Heart Circ. Physiol. 283, H315–H323. doi: 10.1152/ajpheart.00022.2002, PMID: 12063304

[ref61] Niwa K. Younkin L. Ebeling C. Turner S. K. Westaway D. Younkin S.. (2000). A1-40-related reduction in functional hyperemia in mouse neocortex during somatosensory activation. Proc. Natl. Acad. Sci. U. S. A. 97, 9735–9740. doi: 10.1073/pnas.97.17.9735, PMID: 10944232 PMC16934

[ref62] Nortley R. Korte N. Izquierdo P. Hirunpattarasilp C. Mishra A. Jaunmuktane Z.. (2019). Amyloid *β* oligomers constrict human capillaries in Alzheimer’s disease via signaling to pericytes. Science 365:aav9518. doi: 10.1126/science.aav9518, PMID: 31221773 PMC6658218

[ref63] Oakley H. Cole S. L. Logan S. Maus E. Shao P. Craft J.. (2006). Intraneuronal *β*-amyloid aggregates, neurodegeneration, and neuron loss in transgenic mice with five familial Alzheimer’s disease mutations: potential factors in amyloid plaque formation. J. Neurosci. 26, 10129–10140. doi: 10.1523/JNEUROSCI.1202-06.2006, PMID: 17021169 PMC6674618

[ref64] Oblak A. L. Lin P. B. Kotredes K. P. Pandey R. S. Garceau D. Williams H. M.. (2021). Comprehensive evaluation of the 5XFAD mouse model for preclinical testing applications: a MODELAD study. Front. Aging Neurosci. 13:713726. doi: 10.3389/fnagi.2021.713726, PMID: 34366832 PMC8346252

[ref65] Park J. C. Baik S. H. Han S. H. Cho H. J. Choi H. Kim H. J.. (2017). Annexin A1 restores A*β*1-42-induced blood–brain barrier disruption through the inhibition of RhoA-ROCK signaling pathway. Aging Cell 16, 149–161. doi: 10.1111/acel.12530, PMID: 27633771 PMC5242298

[ref66] Park L. Hochrainer K. Hattori Y. Ahn S. J. Anfray A. Wang G.. (2020). Tau induces PSD95nNOS uncoupling and neurovascular dysfunction independent of neurodegeneration. Nat. Neurosci. 23, 1079–1089. doi: 10.1038/s41593-020-0686-7, PMID: 32778793 PMC7896353

[ref67] Park L. Koizumi K. El Jamal S. Zhou P. Previti M. L. Van Nostrand W. E.. (2014). Age dependent neurovascular dysfunction and damage in a mouse model of cerebral amyloid angiopathy. Stroke 45, 1815–1821. doi: 10.1161/STROKEAHA.114.005179, PMID: 24781082 PMC4284427

[ref68] Profaci C. P. Munji R. N. Pulido R. S. Daneman R. (2020). The blood-brain barrier in health and disease: important unanswered questions. J. Exp. Med. 217:217. doi: 10.1084/jem.20190062 PMC7144528 32211826

[ref69] Qin L. Wu X. Block M. L. Liu Y. Breese G. R. Hong J.-S.. (2007). Systemic LPS causes chronic neuroinflammation and progressive neurodegeneration. Glia 55, 453–462. doi: 10.1002/glia.20467, PMID: 17203472 PMC2871685

[ref70] Ries M. Watts H. Mota B. C. Lopez M. Y. Donat C. K. Baxan N.. (2021). Annexin A1 restores cerebrovascular integrity concomitant with reduced amyloid-*β* and tau pathology. Brain 144, 1526–1541. doi: 10.1093/brain/awab050, PMID: 34148071 PMC8262982

[ref71] Ronneberger O. Fischer P. Brox T. (2015). “U-net: convolutional networks for biomedical image segmentation,” in Medical Image Computing and Computer-Assisted Intervention – MICCAI 2015. eds. Navab N. Hornegger J. Wells W. Frangi A. *vol.* 9351 (New York: Springer International Publishing), 234–241.

[ref72] Ryu J. K. McLarnon J. G. (2009). A leaky blood-brain barrier, fibrinogen infiltration and microglial reactivity in inflamed Alzheimer’s disease brain. J. Cell. Mol. Med. 13, 2911–2925. doi: 10.1111/j.1582-4934.2008.00434.x 18657226 PMC4498946

[ref73] Sadeghian H. Lacoste B. Qin T. Toussay X. Rosa R. Oka F.. (2018). Spreading depolarizations trigger caveolin-1–dependent endothelial transcytosis. Ann. Neurol. 84, 409–423. doi: 10.1002/ana.25298, PMID: 30014540 PMC6153037

[ref74] Sagare A. P. Bell R. D. Zhao Z. Ma Q. Winkler E. A. Ramanathan A.. (2013). Pericyte loss influences Alzheimer-like neurodegeneration in mice. Nat. Commun. 4:2932. doi: 10.1038/ncomms3932, PMID: 24336108 PMC3945879

[ref75] Sasaguri H. Nilsson P. Hashimoto S. Nagata K. Saito T. De Strooper B.. (2017). APP mouse models for Alzheimer’s disease preclinical studies. EMBO J. 36, 2473–2487. doi: 10.15252/embj.201797397, PMID: 28768718 PMC5579350

[ref76] Saunders N. R. Dziegielewska K. M. Møllgard K. Habgood M. D. (2015). Markers for blood-brain° barrier integrity: how appropriate is Evans blue in the twenty-first century and what are the alternatives? Front. Neurosci. 9:385. doi: 10.3389/fnins.2015.00385, PMID: 26578854 PMC4624851

[ref77] Schindelin J. Arganda-Carreras I. Frise E. Kaynig V. Longair M. Pietzsch T.. (2012). Fiji: an open-source platform for biological-image analysis. Nat. Methods 9, 676–682. doi: 10.1038/nmeth.2019, PMID: 22743772 PMC3855844

[ref78] Seabold S. Perktold J. (2010). Statsmodels: econometric and statistical modeling with python. In Proceedings of the 9th Python in Science Conference (SciPy). Austin, Texas.

[ref79] Selkoe D. J. Hardy J. (2016). The amyloid hypothesis of Alzheimer’s disease at 25 years. EMBO Mol. Med. 8, 595–608. doi: 10.15252/emmm.201606210, PMID: 27025652 PMC4888851

[ref80] Shabir O. Pendry B. Lee L. Eyre B. Sharp P. S. Rebollar M. A.. (2022). Assessment of neurovascular coupling and cortical spreading depression in mixed mouse models of atherosclerosis and Alzheimer’s disease. elife 11:e68242. doi: 10.7554/eLife.68242, PMID: 35014950 PMC8752088

[ref81] Shin H. K. Jones P. B. Garcia-Alloza M. Borrelli L. Greenberg S. M. Bacskai B. J.. (2007). Age-dependent cerebrovascular dysfunction in a transgenic mouse model of cerebral amyloid angiopathy. Brain 130, 2310–2319. doi: 10.1093/brain/awm156 17638859

[ref82] Shorten C. Khoshgoftaar T. M. (2019). A survey on image data augmentation for deep learning. J. Big Data 6, 1–48. doi: 10.1186/s40537-019-0197-0 PMC8287113 34306963

[ref83] Sil A. Erfani A. Lamb N. Copland R. Riedel G. Platt B. (2022). Sex differences in behavior and molecular pathology in the 5XFAD model. J. Alzheimers Dis. 85, 755–778. doi: 10.3233/JAD-210523, PMID: 34864660

[ref84] Sweeney M. D. Kisler K. Montagne A. Toga A. W. Zlokovic B. V. (2018a). The role of brain vasculature in neurodegenerative disorders. Nat. Neurosci. 21, 1318–1331. doi: 10.1038/s41593-018-0234-x, PMID: 30250261 PMC6198802

[ref85] Sweeney M. D. Sagare A. P. Zlokovic B. V. (2018b). Blood-brain barrier breakdown in Alzheimer disease and other neurodegenerative disorders. Nat. Rev. Neurol. 14, 133–150. doi: 10.1038/nrneurol.2017.188, PMID: 29377008 PMC5829048

[ref86] Takano T. Han X. Deane R. Zlokovic B. Nedergaard M. (2007). Two-photon imaging of astrocytic ca2+ signaling and the microvasculature in experimental mice models of Alzheimer’s disease. Ann. N. Y. Acad. Sci. 1097, 40–50. doi: 10.1196/annals.1379.004, PMID: 17413008

[ref87] Tarasoff-Conway J. M. Carare R. O. Osorio R. S. Glodzik L. Butler T. Fieremans E.. (2015). Clearance systems in the brain-implications for Alzheimer disease. Nat. Rev. Neurol. 11, 457–470. doi: 10.1038/nrneurol.2015.119, PMID: 26195256 PMC4694579

[ref88] Tateda K. Matsumoto T. Miyazaki S. Yamaguchi K. (1996). Lipopolysaccharide-induced lethality and cytokine production in aged mice. Infect. Immun. 64, 769–774. doi: 10.1128/iai.64.3.769-774.1996, PMID: 8641780 PMC173836

[ref89] Thomsen M. S. Johnsen K. B. Kucharz K. Lauritzen M. Moos T. (2022). Blood–Brain barrier transport of transferrin Receptor-Targeted nanoparticles. Pharmaceutics 14:2237. doi: 10.3390/pharmaceutics14102237, PMID: 36297671 PMC9608573

[ref90] Tsui K. C. Roy J. Chau S. C. Wong K. H. Shi L. Poon C. H.. (2022). Distribution and inter-regional relationship of amyloid-beta plaque deposition in a 5xFAD mouse model of Alzheimer’s disease. Front. Aging Neurosci. 14:964336. doi: 10.3389/fnagi.2022.964336, PMID: 35966777 PMC9371463

[ref91] Uemura M. T. Maki T. Ihara M. Lee V. M. Y. Trojanowski J. Q. (2020). Brain microvascular pericytes in vascular cognitive impairment and dementia. Front. Aging Neurosci. 12:80. doi: 10.3389/fnagi.2020.00080, PMID: 32317958 PMC7171590

[ref92] Van Rossum G. Drake F. L. (2009). Python 3 Reference Manual: (Python documentation manual part 2) Scotts Valley, CA: CreateSpace Independent Publishing Platform.

[ref93] Virtanen P. Gommers R. Oliphant T. E. Haberland M. Reddy T. Cournapeau D.. (2020). SciPy 1.0: fundamental algorithms for scientific computing in python. Nat. Methods 17, 261–272. doi: 10.1038/s41592-019-0686-2, PMID: 32015543 PMC7056644

[ref94] Wang D. Chen F. Han Z. Yin Z. Ge X. Lei P. (2021). Relationship between amyloid-*β* deposition and blood-brain barrier dysfunction in Alzheimer’s disease. Front. Cell. Neurosci. 15:695479. doi: 10.3389/fncel.2021.695479, PMID: 34349624 PMC8326917

[ref95] Wang H.-M. Huang P. Li Q. Yan L.-L. Sun K. Yan L.. (2019). Post-treatment with QingYing-tang, a compound Chinese medicine relives lipopolysaccharide-induced cerebral microcirculation disturbance in mice. Front. Physiol. 10:1320. doi: 10.3389/fphys.2019.01320, PMID: 31708795 PMC6823551

[ref03] Whitesell J. D. Buckley A. R. Knox J. E. Kuan L. Graddis N. Pelos A.. (2019). Whole brain imaging reveals distinct spatial patterns of amyloid beta deposition in three mouse models of Alzheimer’s disease. J. Comp. Neurol. 527, 2122–2145. doi: 10.1002/cne.24555 30311654 PMC8026112

[ref96] Yang H. S. Onos K. D. Choi K. Keezer K. J. Skelly D. A. Carter G. W.. (2021). Natural genetic variation determines microglia heterogeneity in wild-derived mouse models of Alzheimer’s disease. Cell Rep. 34:108739. doi: 10.1016/j.celrep.2021.108739, PMID: 33567283 PMC7937391

[ref97] Yang A. C. Stevens M. Y. Chen M. B. Lee D. P. Stahli D. Gate D.. (2020). Physiological¨ blood-brain transport is impaired with age by a shift in transcytosis. Nature 583, 425–430. doi: 10.1038/s41586-020-2453-z, PMID: 32612231 PMC8331074

[ref98] Yin X. Zhang X. Zhang J. Yang W. Sun X. Zhang H.. (2022). High-resolution digital panorama of multiple structures in whole brain of Alzheimer’s disease mice. Front. Neurosci. 16:870520. doi: 10.3389/fnins.2022.870520, PMID: 35516801 PMC9067162

[ref99] Zambach S. A. Cai C. Helms H. C. C. Hald B. O. Dong Y. Fordsmann J. C.. (2021). Precapillary sphincters and pericytes at first-order capillaries as key regulators for brain capillary perfusion. Proc. Natl. Acad. Sci. U. S. A. 118:e2023749118. doi: 10.1073/pnas.2023749118, PMID: 34155102 PMC8255959

[ref100] Zhu J. Li Z. Ji Z. Wu Y. He Y. Liu K.. (2022). Glycocalyx is critical for blood-brain barrier integrity by suppressing caveolin1-dependent endothelial transcytosis following ischemic stroke. Brain Pathol. 32:e13006. doi: 10.1111/bpa.13006, PMID: 34286899 PMC8713524

