# Supplementary material for: Preserved blood-brain barrier and neurovascular coupling in female 5xFAD model of Alzheimer’s disease
Source: Front Aging Neurosci. 2023 May 5;15:1089005. doi: 10.3389/fnagi.2023.1089005 (PMC10228387; doi:10.3389/fnagi.2023.1089005)
Supplement: Supplementary file 1 [file Data_Sheet_1.pdf]

Supplementary material for: Preserved blood-brain barrier and neurovascular coupling in female 5xFAD model of Alzheimer's disease

Zhukov O, He C, Soylu-Kucharz R, Cai C, Lauritzen AD, Aldana BI, Björkqvist M, Lauritzen M and Kucharz K

Correspondance:

Martin Lauritzen, mlauritz@sund.ku.dk

Krzysztof Kucharz, kucharz@sund.ku.dk

This PDF file includes:

Supplementary text (sections 1–4)

Table S1

Figures S1–S11

Captions for tables S2, S3, and videos S1–S7

References

1 PLACEMENT OF ROIS IN IMAGES OF BRAIN PARENCHYMA TO QUANTIFY THE PARACELLULAR LEAKAGE OF THE BBB

After i.a. injection of sodium fluorescein (NaFluo), it gradually leaks into the brain parenchyma. The intensity of NaFluo in parenchyma is highly heterogeneous (fig. S1 A). This can lead to a substantial bias of quantification of paracellular leakage (fig. S1 B). To avoid this bias, we classified factors contributing to heterogeneity into groups (table S1). Specifically, intensity is reduced under the pial vessels (shadow from pial vessel) and under amyloid plaques (shadow from plaque). Smaller groups of bright pixels and groups of dark pixels (anomalies) can introduce positive and negative bias on the qiantification of leakage, respectively. Accordingly, for quantification of paracellular leakage, we placed regions of interest (ROIs) at a distance from pial vessels, plaques, and anomalies.

2 ESTIMATE OF AMYLOID PLAQUE VOLUME DENSITY IN THE CEREBRAL CORTEX FROM DATA REPORTED IN GIANNONI ET AL. (2016)

Giannoni et al. (2016) quantified the density of thioflavin S-labeled amyloid-β (Aβ) plaques from two-dimensional images and reported the plaque number per mm^2, i.e., the surface density, for frontal, parietal upper, parietal lateral, and entorhinal cortices (Giannoni et al., 2016, Table 1). Assuming that the density of the plaques is the same in all directions, the volume density of plaques can be estimated from the surface density ρ_s: $\rho_v = \rho_s^{3/2}$. ρ_v calculated from values reported in (Giannoni et al., 2016) ranges from 1013 ± 91 to 2185 ± 222 plaques/mm^3.

3 PARACELLULAR LEAKAGE IN WILD-TYPE MICE

Paracellular leakage of tracer molecules (in our case, NaFluo) is determined by the flux of NaFluo across the blood-brain barrier (BBB) from the blood into the brain parenchyma and by the clearance of NaFluo from the brain parenchyma. The flux into parenchyma depends on the concentration gradient of NaFluo across the BBB, and on the permeability coefficient of the BBB for NaFluo. Paracellular leakage is zero if the flux of NaFluo into parenchyma is zero or smaller than the clearance. Our data shows increase of NaFluo in parenchyma which means that the flux into parenchyma is larger than the clearance during the recording (fig. 4). The flux into parenchyma can be zero only if either the concentration gradient is zero (no dye in the blood) or if the permeability coefficient is zero. The concentration gradient is larger than zero because the dye has been injected into the blood. The permeability coefficient of NaFluo across the BBB equals $(3.91 \pm 0.41) \times 10^{-7}\mathrm{cm/s}$ as determined by (Kutuzov et al., 2018, Fig 4A, F). Therefore, even under control conditions, there is a non-zero flux of NaFluo across the BBB. In addition, studies have shown that the leakage of NaFluo (Mathiesen Janiurek et al., 2019) as well as larger dyes (10-kDa dextran) (Andreone et al., 2017, Fig 2O) across the BBB is non-zero in healthy brain and WT mice.

4 REMOVAL OF OUTLIERS AND TYPE-I ERROR RATE

We avoided using outlier tests because removal of outliers increases the type-I error rate when the compared samples have different variances. Figure 6F shows that in each vessel type, the standard deviation of the mean relative dilation is different between WT and 5xFAD, which suggests different variances of relative dilation in WT and 5xFAD. Removal of suspected outliers will shift the mean of a sample with a larger standard deviation more strongly than the mean of a sample with a smaller standard deviation. As the result, removal of outliers can increase the difference between the means and inflate the type-I error rate, i.e. the

Table S1. Factors affecting the intensity of NaFluo in brain parenchyma

Factor	How affects the intensity
Shadow from amyloid plaque	decreases
Shadow from pial vessel	decreases
Clusters of bright/dark pixels	increases/decreases

probability of rejecting the null hypothesis when it is true. In addition, the relative dilation may naturally have a positive skew as can be seen in data published previously: Figure 3e in (Grubb et al., 2020) and Figure 1g in (Cai et al., 2023). The data published in the presnt study confirms this view (Figure 6F).

FIGURES

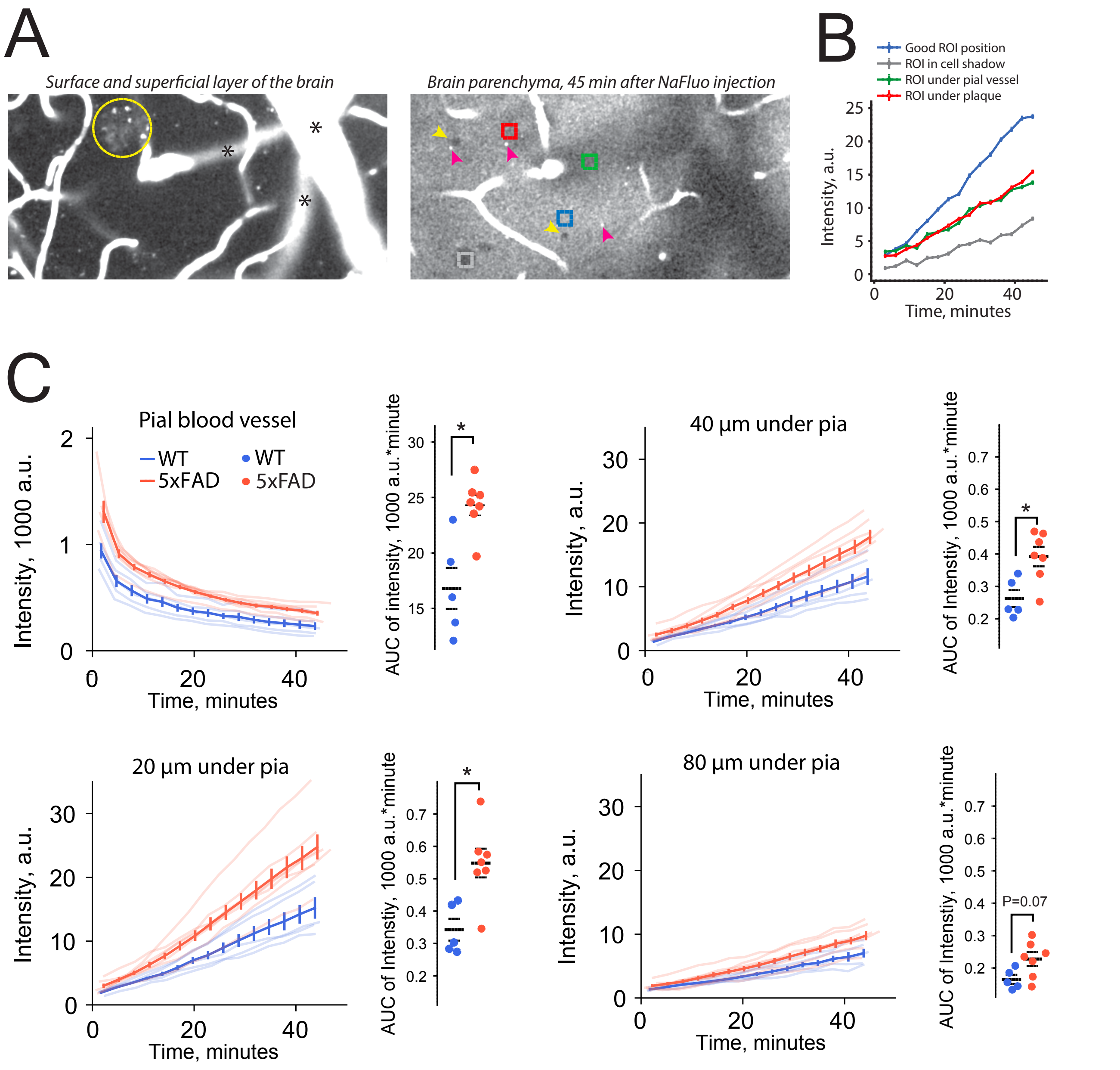

Figure S1. Paracellular leakage across the blood-brain barrier in 5xFAD mice. **(A)** Image of the brain surface and superficial layer of the brain at 2 minutes after injection of NaFluo (left) and of brain parenchyma 40 um under the surface at 45 min after injection of NaFluo. Even though the dye accumulated in parenchyma (right, brighter regions), certain regions remain dark. The dark regions are located right under (a) pial vessels (left, asterisks), (b) plaques (left, yellow circle), suggesting that the shadowing effect is related to absorption of the fluorescence light by blood and by plaques. "Shadowed regions" as well as other inhomogenities of the parenchyma intensity (right, arrowheads) were avoided when when placing the ROIs for averaging the parenchyma signal. Images were average intensity projections over three focal planes. **(B)** Intensity within ROIs placed at different locations in parenchyma: in a shadowed region (red, green), in a uniform bright region (blue), in other inhomogeneity (gray). Bad placement of ROIs (inside "shadowed" region or in small inhomogeneity) results in up to two-fold underestimation of the intensity in parenchyma (compare blue and gray curves at 40 minutes). **(C)**. Intensity of NaFluo in the blood of a pial vessel (top left) and in the brain parenchyma at specified depth (other) in 5xFAD and WT 5xFAD mice. The time graph shows the change of NaFluo intensity over time, and the dot plots show the area under the curve (AUC) of the intensity in the corresponding graphs (two-tailed Mann-Whitney U test-test, * $P<0.05$, n(WT)=5, n(5xFAD)=7).

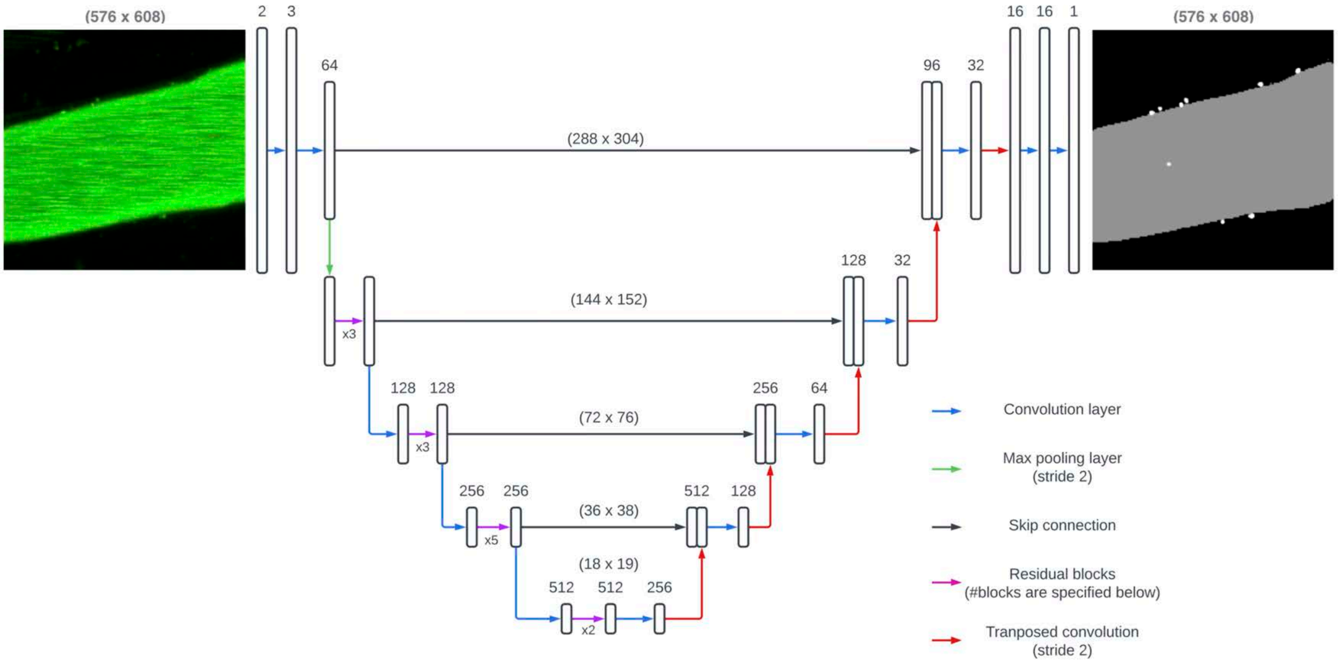

Figure S2. Architecture of the U-net neural network trained to detect albumin punctae and pial vessels. The encoding part is a often used and thoroughly-validated ResNet34 model initiated with weights trained on the ImageNet challenge of natural images to support better and faster convergence considering the relatively small training dataset (He et al., 2016; Russakovsky et al., 2014). Each box represents a feature map and the number of channels in each feature map is specified above the boxes. The sizes of the feature maps are specified in parentheses for each level of the U-net. The different layers in the model are color coded arrows and the types of layers are specified in the lower right corner. The input to a model is a 2-channel ROI from a two-photon microscopy image of a pial brain vessel. The output of the vessel-detecting model (Model-1) is a vessel mask; the output of the punctae-detecting model (Model-2) is a punctae mask. In this diagram, punctae and vessel masks are overlayed for simplicity. The two models have been trained separately to recognize punctae and vessels. Both models were trained to optimize the soft Dice coefficient (DICE) coefficient for 200 epochs with a learning rate of 0.0001 using data augmentation. In order to create a binary segmentation mask from the continuous confidence scores outputted by the model, the optimal threshold was estimated in the validation dataset. This optimal threshold was applied when generating masks for data from 5xFAD mice.

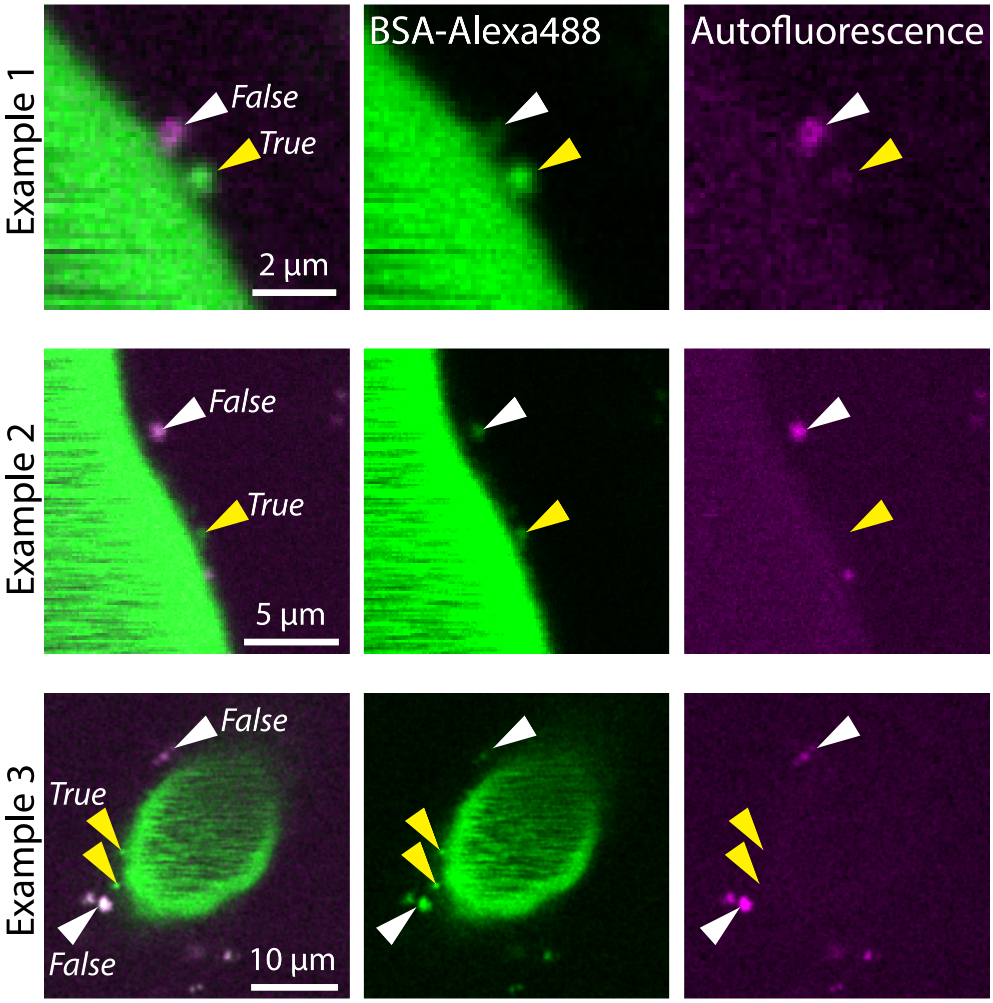

Figure S3. AMT was quantified by counting BSA-positive punctae but not autofluorescent punctae. During image acquisition for AMT measurement, two channels were recorded: BSA-Alexa and autofluorescence. A punctum was counted if its fluorescence was dominated by BSA-Alexa (True, yellow arrowheads). A punctum was not counted if its fluorescence was dominated by autofluorescence (False, white arrowheads). Images are single focal planes (top, bottom), and maximum intensity projection of three focal planes separated by $2.5\ \mu$m (middle); three rows show three different vessels.

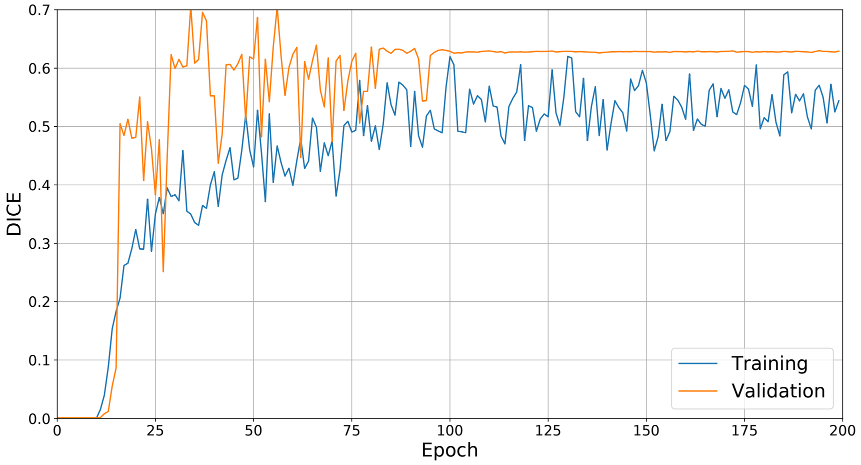

Figure S4. Training and validation curves of Model-2, showing the DICE coefficient at each epoch. One epoch is a complete iteration of the whole training dataset. During training, the data was heavily augmented while the validation data was not augmented. Consequently, the DICE at convergence is lower in the training than in the validation.

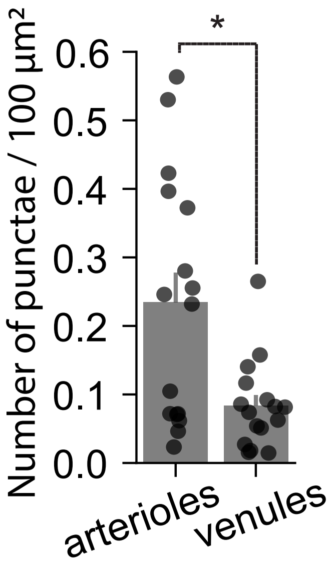

Figure S5. Adsorptive-mediated transcytosis (AMT) was increased in arterioles versus venules. Due to the absence of AMT difference between the genotypes, the data was pulled together to gain power in comparison of arteriolar and venular parts of the vasculature (Mann-Whitney U test-test, * $P<0.05$).

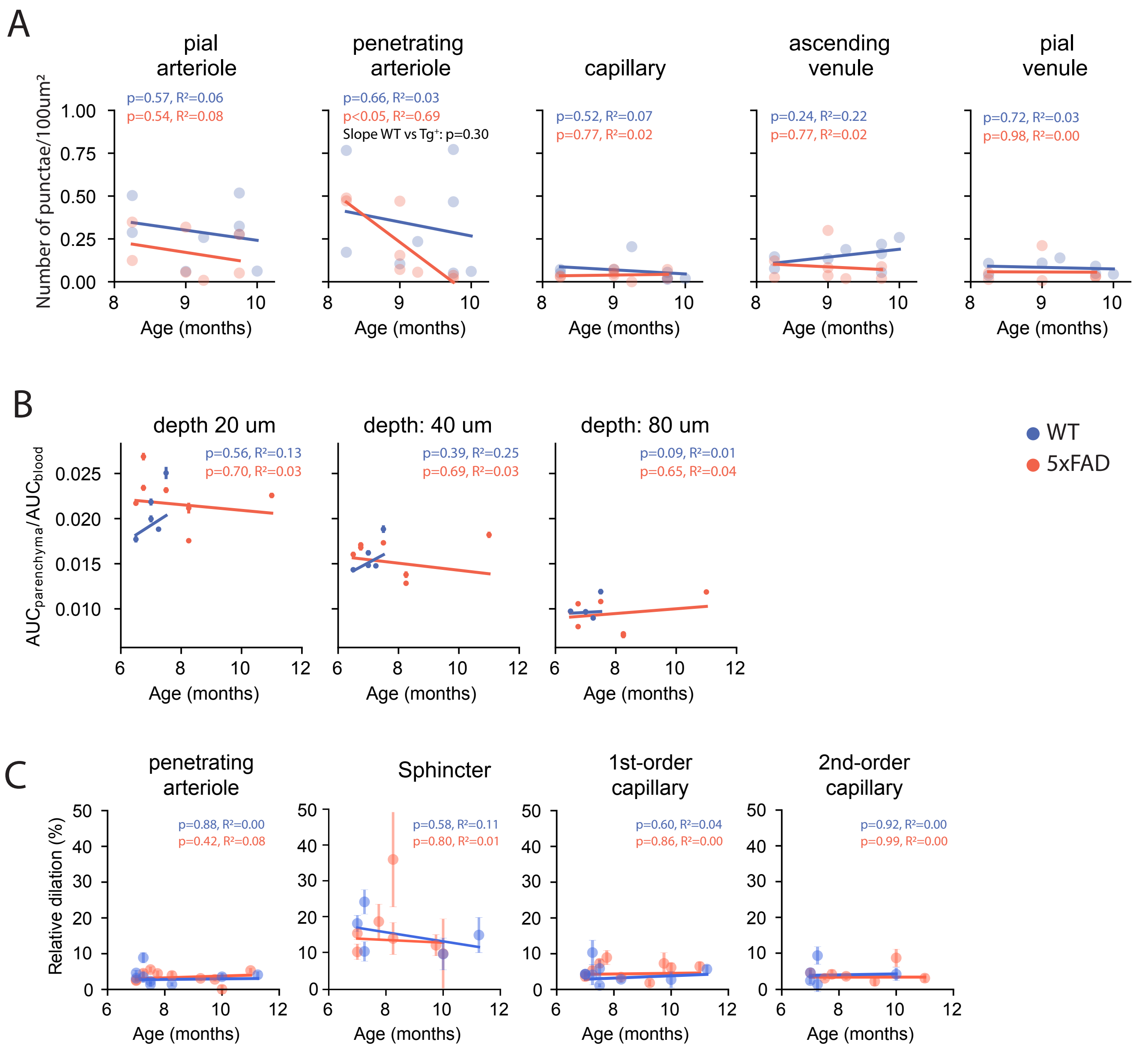

Figure S6. Regression of AMT (A), paracellular leakage (B), and NVC (C) against mouse age suggests no difference between WT and 5xFAD. Data in (A) was fitted with ordinary least-squares; data in (B) and (C) was fitted with weighted least-squares. Only those data were fitted where the age span was no less than one month. At the significance level 0.05, the regression suggested no significant slope of the variables against the age, except AMT in penetrating arterioles in 5xFAD mice. To optimally use all data to compare the AMT between 5xFAD and WT, we compared the slope of AMT versus age between the genotypes. Multivariate regression (the first factor is age, the second factor is the genotype) showed that there was no significant difference between the slopes ((A), *penetrating arteriole*, p=0.30), suggesting no difference between the genotypes. In each panel, one dot represents one mouse. The color-coded p-values were obtained by testing the null hypothesis that the slope is equal to zero.

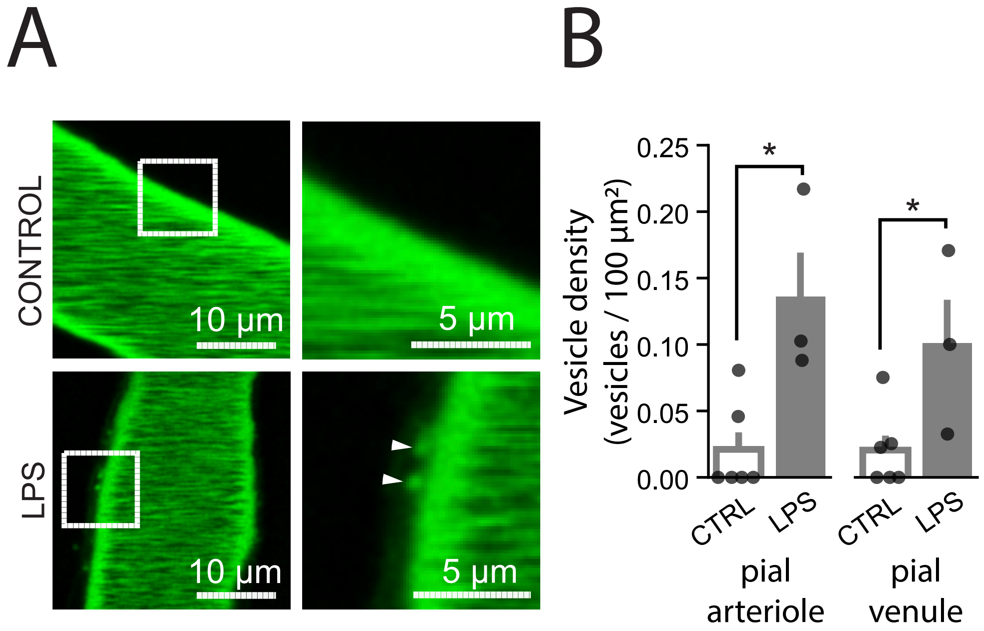

Figure S7. Adsorptive-mediated transcytosis (AMT) was increased in lipopolysaccharide (LPS) model of inflammation. **(A)** Representative images of pial vessels of mice treated with LPS and the control mice. **(B)** Manual quantification of AMT in pial arterioles and venules (Mann-Whitney U test, * $P<0.05$, n(CONTROL)=6, n(LPS)=3).

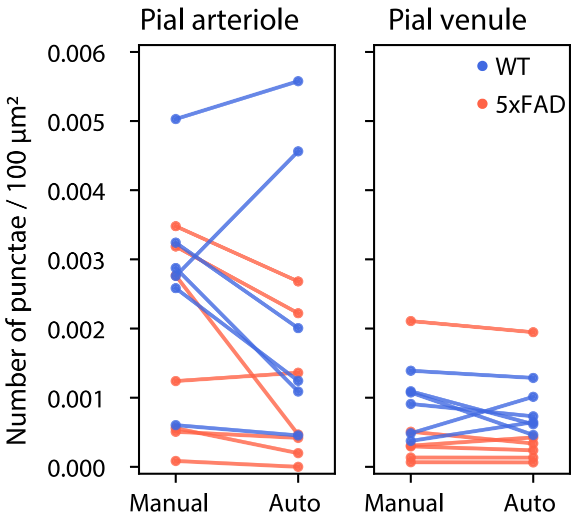

Figure S8. Comparison of punctae density estimated by manual and automated counting. One dot corresponds to a single mouse. The estimates from the same mouse are connected by lines. Although the estimates of punctae density in individual mice may diverge between automated and manual quantification, the average estimates are consistent between the two methods (Figure 3). In some datasets, the smaller density estimated by the machine learning approach is attributable to the difficulty of recognition of small objects (punctae) on a complex background of fluorescence of blood plasma and autofluorescence of the brain parenchyma. The number of mice is smaller than in Figure 3 because some of the datasets used for manual analysis were also used in the training of the models, and hence could not be used for quantification with the automated method.

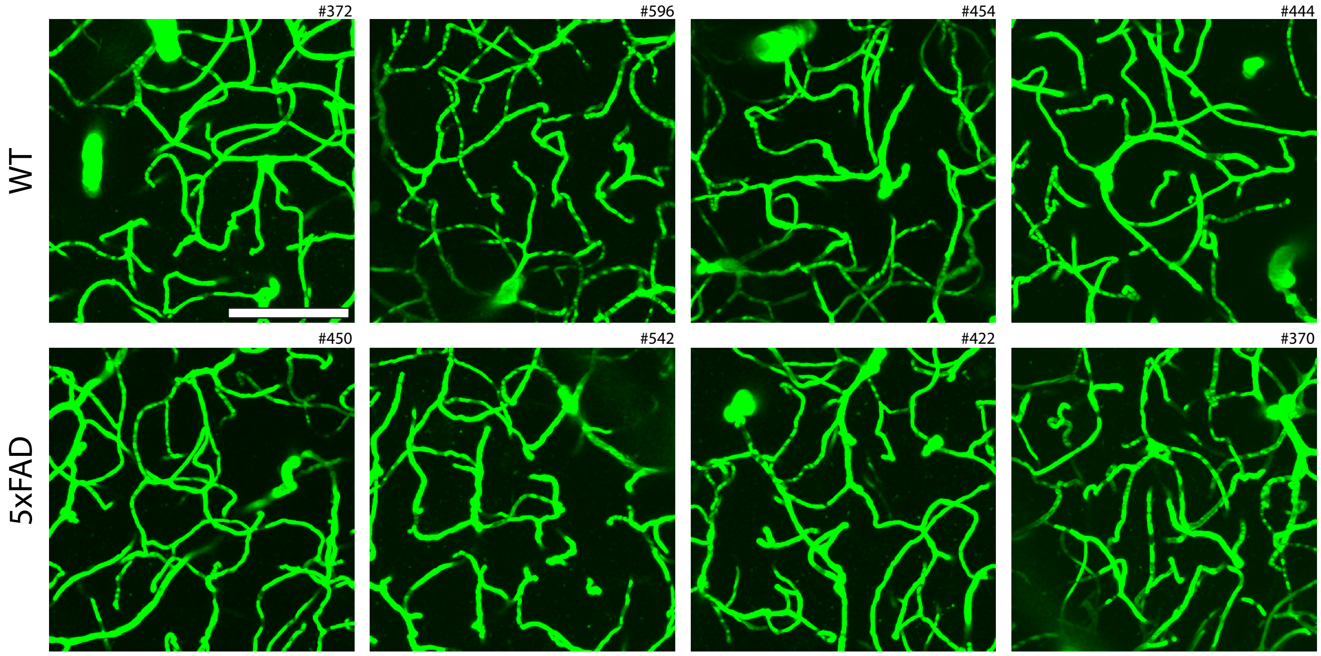

Figure S9. Projections of in vivo 2PM Z-stacks (70–150 μm bellow brain surface) from WT (top) and 5xFAD (bottom) mice. Variable intensity of capillaries (e.g. capillaries in the bottom-left corners of #596 and #370 are darker than other capillaries from the same mice) can be explained by presence of pial vessels (not shown) above the capillaries as illustrated in figure S1A. The range of intensities was saturated for clearer visualization of the vessels. Scale bar 100 μm.

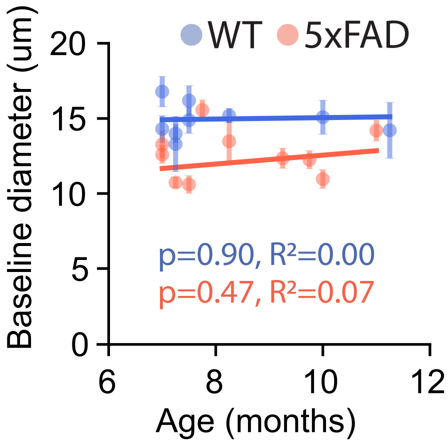

Figure S10. Baseline diameter of penetrating arteriole versus mouse age. One dot represents one mouse. Error bars are SEM. Solid lines show weighted least-squares regressions. The p-values were obtained by testing the null hypothesis that the slope is equal to zero.

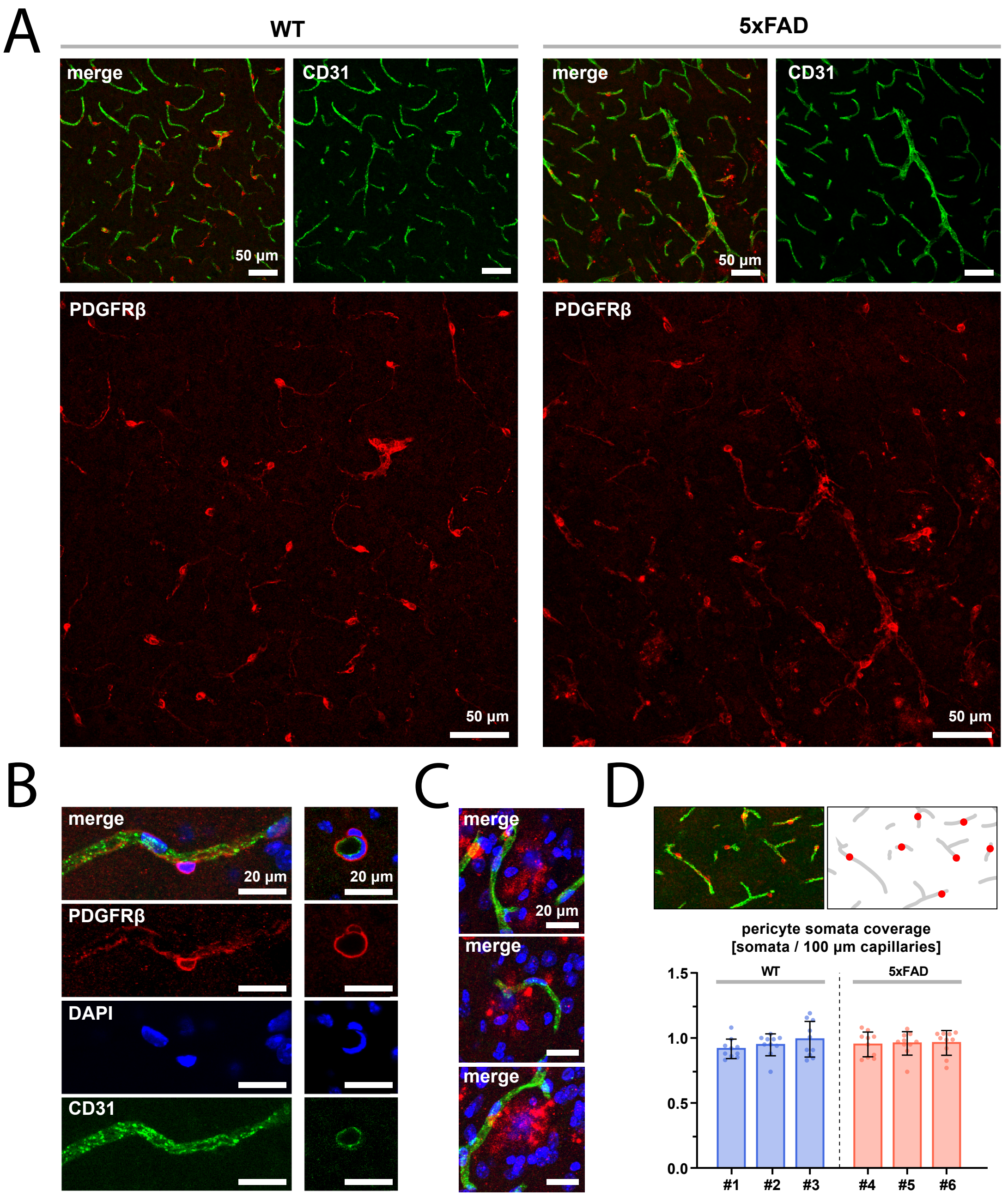

Figure S11. No overt loss of capillary pericytes in 5xFAD mice. (A) Representative confocal microscopy images showing endothelium immunostained for CD31 (green) and pericytes PDGFR-β immunostaining (red) in the cortex. The magnified panel shows a relatively high density of PDGFR-β -positive pericytes in both WT and 5xFAD mice. (B) An example of a capillary pericyte with a typical "bump on a log" morphology and processes extending along the microvessel. Left panels show a capillary oriented perpendicular to the imaging plane; right panels show a cross-section of a capillary. (C) Examples of Aβ plaques (visible on red/PDGFR-β channel) in proximity to capillaries. The plaques were absent in WT mice. (D)The upper panels illustrate the principle of quantification of the pericyte density, i.e., the number of capillary pericyte somata (red) per total length of associated capillaries (gray). Lower panels show no apparent loss of pericytes in 5xFAD mice, with highly consistent pericyte density within and between the genotypes. The data is presented as an average with standard deviation (STD) for each analyzed animal (demarked with #), with data points illustrating individual sampled volumes (Z-stacks) used for pericyte quantification. All images were obtained from Z-stacks following maximum-intensity projection.

LEGENDS FOR SUPPLEMENTARY VIDEOS AND TABLES

Table S2.

Descriptive and test statistics.

Table S3.

Studies quantifying BBB and NVC in various mouse models of AD.

Video 1.

Z-stacks showing autofluorescence of the brain of WT (left) and 5xFAD (right) mice. The depth (top-left corner) is indicated relative to approximate brain surface level. The yellow arrows point to amyloid plaques. No plaques are observed in the WT brain.

Video 2.

Z-stack showing Aβ plaques labeled with thioflavin-S.

Video 3.

Projected hyperstack showing a gradual appearance of BSA-Alexa488-positive punctae at the BBB interface *in vivo*. Time is relative to the time of the BSA-Alexa488 injection.

Video 4.

Movement of BSA punctae at the BBB interface. Circulating BSA labels the plasma inside the blood vessels on the brain surface. The BSA punctae (bright dots) move along the vessel boundary. The time-lapse images were collected from a single focal plane at 3–4 hours after a bolus injection of BSA.

Video 5.

Movement of BSA punctae inside an endothelial cell of a pial arteriole. BSA is present in the blood vessel lumen (bottom) and on the abluminal side of the vessel (top). The images were collected from a single focal plane at 3–4 hours after BSA injection.

Video 6.

Time-lapse recording of paracellular leakage (paracellular leakage) of NaFluo into the brain parenchyma. Time is relative to the time of the NaFluo injection.

Video 7.

Time-lapse recording of vasodilation of penetrating arteriole and associated capillaries upon whisker-pad stimulation (“STIM 2Hz”).

REFERENCES

Andreone, B. J., Chow, B. W., Tata, A., Lacoste, B., Ben-Zvi, A., Bullock, K., et al. (2017). Blood-Brain barrier permeability is regulated by lipid Transport-Dependent suppression of Caveolae-Mediated transcytosis. *Neuron* 94, 581–594.e5. doi:10.1016/j.neuron.2017.03.043

Cai, C., Zambach, S. A., Grubb, S., Tao, L., He, C., Lind, B. L., et al. (2023). Impaired dynamics of precapillary sphincters and pericytes at first-order capillaries predict reduced neurovascular function in the aging mouse brain. *Nature Aging* , 1–12doi:10.1038/s43587-022-00354-1

Giannoni, P., Arango-Lievano, M., Neves, I. D., Rousset, M. C., Baranger, K., Rivera, S., et al. (2016). Cerebrovascular pathology during the progression of experimental alzheimer's disease. *Neurobiol. Dis.* 88, 107–117

Grubb, S., Cai, C., Hald, B. O., Khennouf, L., Murmu, R. P., Jensen, A. G. K., et al. (2020). Precapillary sphincters maintain perfusion in the cerebral cortex. *Nat. Commun.* 11

He, K., Zhang, X., Ren, S., and Sun, J. (2016). Deep residual learning for image recognition. In *2016 IEEE Conference on Computer Vision and Pattern Recognition (CVPR)*. 770–778. doi:10.1109/CVPR.2016.90

Kutuzov, N., Flyvbjerg, H., and Lauritzen, M. (2018). Contributions of the glycocalyx, endothelium, and extravascular compartment to the blood–brain barrier. *Proc. Natl. Acad. Sci. U. S. A.* 115, E9429–E9438. doi:10.1073/pnas.1802155115

Mathiesen Janiurek, M., Soylu-Kucharz, R., Christoffersen, C., Kucharz, K., and Lauritzen, M. (2019). Apolipoprotein m-bound sphingosine-1-phosphate regulates blood-brain barrier paracellular permeability and transcytosis. *Elife* 8, 1–22

Nehra, G., Bauer, B., and Hartz, A. M. S. (2022). Blood-brain barrier leakage in alzheimer's disease: From discovery to clinical relevance. *Pharmacol. Ther.* 234, 108119. doi:10.1016/j.pharmthera.2022.108119

Niwa, K., Younkin, L., Ebeling, C., Turner, S. K., Westaway, D., Younkin, S., et al. (2000). A1-40-related reduction in functional hyperemia in mouse neocortex during somatosensory activation. *Proc. Natl. Acad. Sci. U. S. A.* 97, 9735–9740

Oblak, A. L., Lin, P. B., Kotredes, K. P., Pandey, R. S., Garceau, D., Williams, H. M., et al. (2021). Comprehensive evaluation of the 5XFAD mouse model for preclinical testing applications: A MODEL-AD study. *Front. Aging Neurosci.* 13, 713726. doi:10.3389/fnagi.2021.713726

Park, L., Hochrainer, K., Hattori, Y., Ahn, S. J., Anfray, A., Wang, G., et al. (2020). Tau induces PSD95-nNOS uncoupling and neurovascular dysfunction independent of neurodegeneration. *Nat. Neurosci.* doi:10.1038/s41593-020-0686-7

[Dataset] Russakovsky, O., Deng, J., Su, H., Krause, J., Satheesh, S., Ma, S., et al. (2014). Imagenet large scale visual recognition challenge. doi:10.48550/ARXIV.1409.0575

Whitesell, J. D., Buckley, A. R., Knox, J. E., Kuan, L., Graddis, N., Pelos, A., et al. (2019). Whole brain imaging reveals distinct spatial patterns of amyloid beta deposition in three mouse models of alzheimer's disease. *J. Comp. Neurol.* 527, 2122–2145. doi:10.1002/cne.24555
