# Supplementary material for: Preserved blood-brain barrier and neurovascular coupling in female 5xFAD model of Alzheimer’s disease
Source: Front Aging Neurosci. 2023 May 5;15:1089005. doi: 10.3389/fnagi.2023.1089005 (PMC10228387; doi:10.3389/fnagi.2023.1089005)
Supplement: Supplementary file 2 [file Table_2.pdf]

Table S2

Experiment	Variable	Units	subtyp	grp1	grp2	N1	N2	Mean1	Mean2	SEM1	SEM2
AMT	Punctae density	#punctae/100um2	penetrating arteriole (brain)	WT	5xFAD	6	8	0.3168489886119158	0.2880711390584348	0.1052691686096719	0.1027871397867911
AMT	Punctae density	#punctae/100um2	penetrating arteriole (pia)	WT	5xFAD	6	7	0.2639423096900546	0.1840719750982948	0.1081923097585446	0.0618277445158099
AMT	Punctae density	#punctae/100um2	pial arteriole	WT	5xFAD	8	7	0.286186025234475	0.1689066745839592	0.0564687315947074	0.0496213121131823
AMT	Punctae density	#punctae/100um2	capillary	WT	5xFAD	8	8	0.0635336410866916	0.0392046012117723	0.0203156385052637	0.0082910266589529
AMT	Punctae density	#punctae/100um2	ascending venule (brain)	WT	5xFAD	5	7	0.2012966008247198	0.1405261810837411	0.0175458688808193	0.0440311154065246
AMT	Punctae density	#punctae/100um2	ascending venule (pia)	WT	5xFAD	7	8	0.1245671320690769	0.054088488639397	0.0275596867877731	0.0168636614550981
AMT	Punctae density	#punctae/100um2	pial venule	WT	5xFAD	7	6	0.0823667436718317	0.0569659235465864	0.0137624309676872	0.0286657661514231
AMT	Punctae density	#punctae/100um2	all mice	Arterioles	Venules	8	8	0.0988532029931765	0.0684703811284906	0.0120753335327182	0.0276975852940507
AMT	Punctae density	#punctae/100um2	Arterioles	LPS	CONTROL	3	6	0.1358768467252129	0.0210696738656118	0.033292099550075	0.012836273693452
AMT	Punctae density	#punctae/100um2	Venules	LPS	CONTROL	3	6	0.1011230510071795	0.0205262062362563	0.0325568716715779	0.0109068263401166
AMT	Punctae density	#punctae/100um2	Arteriole/WT	Manual	Automatic	8	7	0.0028618602523447	0.0023406929438367	0.000564687315947	0.0006799328517828
AMT	Punctae density	#punctae/100um2	Arteriole/5xFAD	Manual	Automatic	7	8	0.0016890667458395	0.0010501554573438	0.0004962131211318	0.000323471935931
AMT	Punctae density	#punctae/100um2	Venule/WT	Manual	Automatic	7	8	0.0008236674367183	0.0008050433998353	0.0001376243096768	0.0001040544333716
AMT	Punctae density	#punctae/100um2	Venule/5xFAD	Manual	Automatic	6	8	0.0005696592354658	0.0004177506938168	0.0002866576615142	0.0002087715749903
NVC	Baseline diameter	um	penetrating arteriole	WT	5xFAD	9	10	14.823738696488697	12.59777601010101	0.3665351675489332	0.4784147986183571
NVC	Baseline diameter	um	Sphincter	WT	5xFAD	7	8	4.012103174603174	3.7033125	0.2805867153232571	0.2127190718936393
NVC	Baseline diameter	um	1st-order capillary	WT	5xFAD	9	10	5.927576719576719	5.132158333333333	0.4022984374375932	0.2036680821861612
NVC	Baseline diameter	um	2nd-order capillary	WT	5xFAD	5	9	5.297833333333333	4.749407407407408	0.2135183572644024	0.2255146151361235
NVC	Relative dilation	%	penetrating arteriole	WT	5xFAD	9	10	3.700405603655604	3.486548737373737	0.7012157543022527	0.4891165631505808
NVC	Relative dilation	%	Sphincter	WT	5xFAD	7	8	17.417087301587305	17.693098214285712	2.131644497099069	3.0060267320497056
NVC	Relative dilation	%	1st-order capillary	WT	5xFAD	9	10	4.57762962962963	5.449997222222222	0.820030521437471	0.6521249484466716
NVC	Relative dilation	%	2nd-order capillary	WT	5xFAD	5	9	4.461	4.212444444444445	1.2406245604533224	0.6034326953626622
BBB leakage	AUC_NaFluo	a.u. * min	vessel	WT	5xFAD	5	7	16808.001099981826	24306.88725243393	1747.2041311088349	834.3178903589792
BBB leakage	AUC_NaFluo	a.u. * min	parenchyma (depth: 20 um)	WT	5xFAD	5	7	342.63157262563703	548.3675429999828	30.83745787403353	40.53011877913156
BBB leakage	AUC_NaFluo	a.u. * min	parenchyma (depth: 40 um)	WT	5xFAD	5	7	262.2983999866247	391.99528338142807	23.732022809423928	26.84754777012207
BBB leakage	AUC_NaFluo	a.u. * min	parenchyma (depth: 80 um)	WT	5xFAD	5	7	164.91794750511647	227.76491908494916	12.138406630448268	19.328899032837946
BBB leakage	AUC_NaFluo_parenchyma/AUC_NaFluo_vessel	unitless	parenchyma (depth: 80 um)	WT	5xFAD	5	7	0.020679662206595	0.0223462394659023	0.0011536381569153	0.0009807079771527
BBB leakage	AUC_NaFluo_parenchyma/AUC_NaFluo_vessel	unitless	parenchyma (depth: 80 um)	WT	5xFAD	5	7	0.0157904126656608	0.0160060326954696	0.0007364207406123	0.0006897084071254
BBB leakage	AUC_NaFluo_parenchyma/AUC_NaFluo_vessel	unitless	parenchyma (depth: 80 um)	WT	5xFAD	5	7	0.0099895715227778	0.0093220140456595	0.0004451181112031	0.0006631854264074
NVC	LFP amplitude	mV	negative peak	WT	5xFAD	7	8	-1.1458148808192827	-1.0335494354644612	0.2460832576970152	0.0755457510890615

Table S2

Median1	Median2	25-percentile1	25-percentile2	75-percentile1	75-percentile2	Shapiro_1_Statistic	Shapiro_2_Statistic	Shapiro_1_P	Shapiro_2_P
0.2381445121102406	0.1408861509621595	0.1016048498905391	0.0751420053266062	0.4694318050764505	0.4962313985291521	0.9042233228683472	0.8439137935638428	0.3994952142238617	0.0826084613800048
0.1528790534258733	0.1142060696157999	0.0811711601562092	0.0510111997089408	0.3789639966357637	0.3031453474766656	0.8656212091445923	0.905118465423584	0.2093017101287841	0.3631088435649872
0.2818052611836866	0.1240297249780979	0.2093319510751286	0.0532396173819416	0.3689954332423126	0.2976553244790144	0.898932933807373	0.8699002265930176	0.2826263904571533	0.1852551251649856
0.0528673480733956	0.0352900301151237	0.0209807215153993	0.0257685757492473	0.0713739967989444	0.0558468745894972	0.750889241695404	0.9532267451286316	0.0083553679287433	0.7436747550964355
0.1976419028700042	0.0858904172109527	0.159858319698569	0.0540054659813457	0.2316184203000555	0.2026493727419522	0.9030194282531738	0.8937585353851318	0.4267982840538025	0.2948479354381561
0.1346274961390603	0.0261532486825808	0.0653715986268193	0.0217530497952265	0.1773300806767421	0.0958775607369999	0.9522103667259216	0.8482649326324463	0.7497683763504028	0.0914857909083366
0.0909560767469969	0.0300752583809333	0.0461944650987279	0.017447509227263	0.1083941535640383	0.0456947892569593	0.9002532362937927	0.6799265146255493	0.332516998052597	0.0037719006650149
0.0890237760288893	0.0391038286509547	0.0767766230366013	0.017470800999606	0.122553793136928	0.0760650917110908	0.9424414038658142	0.6880613565444946	0.6352505683898926	0.0016493534203618
0.1025825605987389	0.0	0.0953187618595496	0.0	0.1597877885276392	0.0343672949885327	0.8333114385604858	0.7053501009941101	0.196781575679779	0.0070350882597267
0.1000565448664982	0.011283383356686	0.0663277355229371	0.0	0.1353851134210812	0.024673293610569	0.9998213052749634	0.7713859677314758	0.9744683504104614	0.0319959409534931
0.0028180526118368	0.0014517001433185	0.0020933195107512	0.0011662443768622	0.0036899543324231	0.0032845755371107	0.898932933807373	0.8365811705589294	0.2826263904571533	0.0923084318637847
0.0012402972497809	0.000760863631855	0.0005323961738194	0.0003631388940769	0.0029765532447901	0.0015762005562784	0.869900107383728	0.9040854573249816	0.1852546036243438	0.314285010099411
0.0009095607674699	0.0006877812106398	0.0004619446509872	0.0005883150618463	0.0010839415356403	0.0010554686898365	0.9002530574798584	0.896194338798523	0.3325162827968597	0.2669001519680023
0.0003007525838093	0.0001859464741175	0.0001744750922726	0.0001147379197616	0.0004569478925695	0.0003598095537496	0.6799264550209045	0.6018658876419067	0.0037718978710472	0.0001671982754487
14.885	12.471616161616	13.99	11.281111111111111	15.297272727272729	13.425	0.9627629518508912	0.9489448666572572	0.8267534375190735	0.6560330390930176
3.82	3.640595238095238	3.529861111111111	3.282857142857143	4.3075	4.081	0.9038589596748352	0.977855622768402	0.3549922704696655	0.951525092124939
5.9275	5.1	5.4275	4.871437499999999	6.543333333333333	5.402083333333335	0.962099015712738	0.9379276037216188	0.8200177550315857	0.5301921963691711
5.1875	4.816666666666666	5.164999999999999	4.34	5.535	5.416666666666667	0.9749547243118286	0.9160556793212892	0.9059942960739136	0.3605864346027374
3.49375	3.5058333333333334	2.2975	2.70780303030303	4.2875	4.511	0.8427656292915344	0.9343174695968628	0.0619884058833122	0.4916739463806152
18.075	14.5965	12.548833333333334	11.530714285714286	22.555	20.455	0.8982706069946289	0.8509450554847717	0.3206374943256378	0.0973909720778465
4.261666666666667	5.715000000000001	2.8383333333333334	3.7565972222222217	5.68	7.0803125	0.8993367552757263	0.9764449000358582	0.2481799274682998	0.9433488249778748
4.2625	3.676666666666667	2.4575	3.1220000000000008	4.7	4.63	0.903097927570343	0.7957375645637512	0.4272441565990448	0.0182541776448488
16009.01824951172	24558.466438293457	13734.052313804626	23870.73745651245	19201.580813598637	25333.88028717041	0.9610814452171326	0.910223126411438	0.8154933452606201	0.3974207639694214
303.1187987923622	550.9873254776	283.50203351676464	522.5330461487174	419.3617221593856	579.1737566292286	0.8039743304252625	0.9129188060760498	0.0872744917869567	0.4164535701274872
229.42480413615704	395.4253634691238	227.83098506927493	363.2419809550047	311.1862200737	449.9135203734041	0.8675922155380249	0.9136345982551576	0.2568104267120361	0.4216122031211853
155.88708579540253	234.78660249710083	143.99692426621914	196.95889839828016	185.1344709455967	260.553813342005	0.940018892288208	0.973275065422058	0.6660719513893127	0.920989155769348
0.0199686963611863	0.0225688878325048	0.0188246147897066	0.0214332048749008	0.0218399581904419	0.0232836139890388	0.9462583661079408	0.9507599472999572	0.7104505896568298	0.7366124987602234
0.014818130768919	0.0168006590849074	0.0147639091252934	0.0149061723837504	0.0162062813001998	0.0171994163145621	0.8229119777679443	0.8929510116577148	0.1229413151741027	0.2904128134250641
0.009683060626186	0.0096998517763201	0.0096416265276702	0.007630261951493	0.0097374544376047	0.0106847728179698	0.7787384390830994	0.9148104786872864	0.0537604130804538	0.43018239736557
-1.0048150332432102	-1.0226301831759332	-1.1019588615820235	-1.1534040754775186	-0.8140439794262562	-0.8883169781477832	0.7457207441329956	0.9451356530189514	0.0114441690966486	0.6622037887573242

Table S2

Shapiro_1_Signif	Shapiro_2_Signif	TtestT	TtestP	TtestSignif	UtestU	UtestP	UtestSignif
n.s.	n.s.	0.1780242218414116	0.8616738708897156	n.s.	28.0	0.662004662004662	n.s.
n.s.	n.s.	0.6102798629912282	0.5540753498701312	n.s.	24.0	0.7195041960117442	n.s.
n.s.	n.s.	1.433774385449247	0.1752493708537951	n.s.	39.0	0.2318570318570318	n.s.
*	n.s.	1.037160703567623	0.3172438390361652	n.s.	36.0	0.7209013209013208	n.s.
n.s.	n.s.	1.0241456275464087	0.329902757326267	n.s.	25.0	0.2676767676767677	n.s.
n.s.	n.s.	2.0857054017728984	0.0572782425443436	n.s.	43.0	0.0938616938616938	n.s.
n.s.	*	0.7681200487171546	0.4585885950937133	n.s.	32.0	0.1375291375291375	n.s.
n.s.	*	0.94059767640137	0.3628583433644609	n.s.	51.0	0.0498834498834498	*
n.s.	*	3.4059557851225284	0.0113501001085862	*	18.0	0.0218895181205661	*
n.s.	*	2.565048286678335	0.0372760946784599	*	17.0	0.0488846339161811	*
n.s.	n.s.	0.553293517162784	0.5894517014796572	n.s.	34.0	0.5358197358197359	n.s.
n.s.	n.s.	1.027600267381848	0.3228791944733135	n.s.	38.0	0.280963480963481	n.s.
n.s.	n.s.	0.1019056440824402	0.9203866381156768	n.s.	26.0	0.8665112665112666	n.s.
*	*	0.4064271627806154	0.6915834600886374	n.s.	31.0	0.4135864135864136	n.s.
n.s.	n.s.	3.437316936662385	0.0031432827528037	*	79.0	0.0062330349697337	*
n.s.	n.s.	0.8277783353897858	0.4227322686970698	n.s.	33.0	0.6125874125874127	n.s.
n.s.	n.s.	1.718210671412771	0.1039156311633849	n.s.	66.0	0.0941663754378797	n.s.
n.s.	n.s.	1.48522686521677	0.1632738172095303	n.s.	33.0	0.1898101898101898	n.s.
n.s.	n.s.	0.2403515639260786	0.8129321938280849	n.s.	42.0	0.8382564863858263	n.s.
n.s.	n.s.	-0.0679420591162351	0.946865639523308	n.s.	29.0	0.9550893550893552	n.s.
n.s.	n.s.	-0.7948651029154007	0.4376491630203829	n.s.	33.0	0.3477455989106975	n.s.
n.s.	*	0.1872349916936436	0.8546048106589719	n.s.	23.0	1.0	n.s.
n.s.	n.s.	-3.853930897163203	0.003191687395381	*	1.0	0.005050505050505	*
n.s.	n.s.	-3.440988304874143	0.0063209311895944	*	2.0	0.0101010101010101	*
n.s.	n.s.	-3.151475822608808	0.0103070674780047	*	3.0	0.0176767676767676	*
n.s.	n.s.	-2.2888262960287062	0.0451036414880661	*	6.0	0.0732323232323232	n.s.
n.s.	n.s.	-1.00378751307432	0.3391514380453531	n.s.	12.0	0.4318181818181819	n.s.
n.s.	n.s.	-0.1917843493950762	0.8517494573898063	n.s.	16.0	0.8762626262626263	n.s.
n.s.	n.s.	0.7002850350719222	0.4997170843530452	n.s.	20.0	0.7550505050505051	n.s.
*	n.s.	-0.4284279939812838	0.6753527169228516	n.s.	30.0	0.8665112665112666	n.s.
