# Supplementary material for: Preserved blood-brain barrier and neurovascular coupling in female 5xFAD model of Alzheimer’s disease
Source: Front Aging Neurosci. 2023 May 5;15:1089005. doi: 10.3389/fnagi.2023.1089005 (PMC10228387; doi:10.3389/fnagi.2023.1089005)
Supplement: Supplementary file 3 [file Table_3.pdf]

Table S3

AD model	Sex	Age, months	BBB leakage to small molecules	BBB leakage to big molecules	Neurovascular coupling	Figure	Reference	Link
5xFAD	females	7-11	**Normal**	**Normal**	**Normal**		**This study**	
APP, hTauP301S	male	5-6	Compromised	Compromised			Bien-Ly et al 2015	https://www.sciencedirect.com/science/article/pii/S0896627315008259
APP, hTauP301S	male	15-16	**Normal**	**Normal**			Bien-Ly et al 2015	https://www.sciencedirect.com/science/article/pii/S0896627315008259
J20	male	9-12	Compromised				Skike et al 2018	https://journals.physiology.org/doi/full/10.1152/ajpheart.00570.2017
Tg2576	not specified	6, 12	Compromised	Compromised			Paul et al 2017	https://rupress.org/jem/article/204/8/1999/46988/Fibrin-deposition-accelerates-neurovascular-damage
Tg2576	not specified	8, 12	Compromised				Goldman et al 2018	https://onlinelibrary.wiley.com/doi/10.1111/acel.12818
E3FAD	male	6	**Normal**			Figure 3	Marottoli et al 2017	https://journals.sagepub.com/doi/10.1177/1759091417719201
5xFAD	male	3	Compromised	Compromised		FIgure 1B	Ries et al 2021	https://academic.oup.com/brain/article/144/5/1526/6306802
5xFAD	male	6	**Normal**	**Normal**		Supplementary figure 3E	Ries et al 2021	https://academic.oup.com/brain/article/144/5/1526/6306802
5xFAD	not specified	3	**Normal**	**Normal**			Liu et al 2020	https://www.sciencedirect.com/science/article/abs/pii/S0006291X20303429
5xFAD	not specified	4, 5	Compromised	Compromised			Liu et al 2020	https://www.sciencedirect.com/science/article/abs/pii/S0006291X20303429
5xFAD	not specified	9-10	Compromised	Compromised		Figure 1E,F	Park et al 2017	https://onlinelibrary.wiley.com/doi/10.1111/acel.12530
E3FAD	males and females	18-24	Compromised				Montagne et al 2021	https://www.nature.com/articles/s43587-021-00073-z
J20	males and females	30			**Normal**		Kimbrough et al 2015	https://academic.oup.com/brain/article/138/12/3716/416119
Duch/Iowa	?	2-4			Compromised		Takano et al 2007	https://nyaspubs.onlinelibrary.wiley.com/doi/abs/10.1196/annals.1379.004
5xFAD	males and females	12-14			Compromised	FIgure 1C,D	Mughal et al 2021	https://academic.oup.com/function/article/2/2/zqab010/6146403
Tg-SwDI	males	3, 18, 24			Compromised		Park et al 2014	https://www.ahajournals.org/doi/10.1161/STROKEAHA.114.005179
Tg2576	males	3, 12			Compromised	Figure 2,4,9C	Park et al 2020	https://www.jneurosci.org/content/40/42/8160
Tg2576	males (Tg2123 M)	3-4			Compromised	Figure 2A (SSC stimulation)	Niwa et al 2000	https://www.pnas.org/doi/10.1073/pnas.97.17.9735
Tg2576	females (Tg2123 F)	3-4			Compromised	Figure 3B	Niwa et al 2000	https://www.pnas.org/doi/10.1073/pnas.97.17.9735
ApoE4	males	3-4			Compromised	Figure 2	Koizumi et al 2018	https://www.nature.com/articles/s41467-018-06301-2
APP	males and females	6			Compromised	Figure 1B	Li et al 2021	https://www.frontiersin.org/articles/10.3389/fphys.2021.715446/full
J20	males	6, 12, 24			**Normal**	Figure 4	Duncombe 2017	https://onlinelibrary.wiley.com/doi/pdf/10.1111/nan.12375
Tg2576		8			**Normal**		Shin et al 2007	https://academic.oup.com/brain/article/130/9/2310/290176
Tg2576		19			Compromised		Shin et al 2007	https://academic.oup.com/brain/article/130/9/2310/290176
